# African Medicinal Plants Targeting Triple-Negative Breast Cancer (TNBC): A Systematic Review of Ethnobotanical Surveys, Phytochemistry and Anti-TNBC Studies

**DOI:** 10.3390/ph19081134

**Published:** 2026-07-23

**Authors:** Judith Flore Tchuissang Mbougnia, Peron Bosco Leutcha, Gervais Mouthe Happi, Adedokun Oluwasegun Adekanmi, Mathieu Tene, Epole Ngolle Ntungwe

**Affiliations:** 1 Department of Organic Chemistry, Faculty of Science, University of Yaoundé I, Yaoundé P.O. Box 812, Cameroon; 2 Department of Chemistry, Faculty of Science, University of Maroua, Maroua P.O. Box 814, Cameroon; peron.leutcha@gmail.com; 3 Department of Chemistry, Faculty of Science, Muğla Sıtkı Koçman University, Muğla 48121, Turkey; 4 Higher Technical Teachers Training College, University of Douala, Douala P.O. Box 2701, Cameroon; gervais20022003@yahoo.fr; 5 Research Center for Biosciences & Health Technologies (CBIOS), Universidade Lusófona, 1749-024 Lisboa, Portugal; f7681@ulusofona.pt; 6 Department of Chemistry, Faculty of Science, University of Dschang, Dschang P.O. Box 67, Cameroon; mtene2001@yahoo.fr; 7 Department of Organic and Pharmaceutical, Chemistry, Institut Quimic de Sarrià, Ramon Llull University, 08017 Barcelona, Spain

**Keywords:** triple-negative breast cancer, African pharmacopoeia, multi-drug resistance, Ethnobotany, cytotoxicity

## Abstract

**Background:** Triple-Negative Breast Cancer (TNBC) remains the most aggressive oncological challenge within the African continent, characterized by high molecular heterogeneity, early onset in African women, and the absence of hormonal receptors. Multi-drug resistance (MDR), driven by ATP-binding cassette (ABC) efflux pumps and supported by the persistence of cancer stem cells (CSCs) within the tumor microenvironment, significantly compromises clinical outcomes and traditional treatment efficacy. **Objective:** This systematic review evaluates the ethnobotanical relevance, phytochemical diversity, and toxicity of some medicinal plants from the African pharmacopoeia specifically utilized or investigated for their activity against the TNBC phenotype. **Methods:** Following PRISMA guidelines, a systematic search was executed across PubMed, ScienceDirect, Scopus, Web of Science, and AJOL. The study focuses on original research published between 2011 and 2026 utilizing TNBC-specific models (such as MDA-MB-231, BT-20, HCC1937, or 4T1) and analyses the growth habits, parts used, and traditional administration modes of the selected African plant species, distributed across Western, Central, Southern, and Eastern Africa. Following a rigorous multi-reviewer screening process, a final set of 52 primary articles representing 30 distinct genera and 22 botanical families was selected for qualitative and quantitative synthesis. **Results**: This review identifies plant species actively used or studied in Africa for their anti-TNBC potential, with the most representative botanical families being Asteraceae (13%), Fabaceae (7%), and Annonaceae (7%). These species are traditionally administered through various methods, including decoction, infusion, and mastication, utilizing diverse plant parts such as leaves, stem and root barks. Significant cytotoxic activities against TNBC cell lines (notably MDA-MB-231) were recorded, with IC_50_ values as low as 5.10 ± 0.28 µg/mL for *Catharanthus roseus* and 13.56 µg/mL for *Nauclea pobeguinii*. Furthermore, species such as *Vernonia amygdalina* and *Curcuma longa* demonstrated a capacity to enhance the effectiveness of conventional chemotherapy like Doxorubicin, suggesting a strong potential for chemosensitization and therapeutic synergy in managing resistant breast cancer phenotypes. **Conclusions:** Standardized African phytomedicines represent a promising frontier in overcoming TNBC chemoresistance. However, the lack of clinical trials and end-to-end drug development infrastructure on the continent remains a significant barrier. Transitioning to nano-formulated delivery systems and establishing regional drug discovery hubs under the ethical framework of the Nagoya Protocol are essential steps toward developing precise and equitable oncology treatments from Africa’s rich biodiversity.

## 1. Introduction

Breast cancer is a disease in which abnormal cells in the breast grow out of control and form tumors. If left unchecked, the tumours can spread throughout the body and become fatal. In 2022, there were an estimated 2.3 million women diagnosed with breast cancer and 670,000 deaths globally [1]. Breast cancer occurs in every country of the world in women at any age after puberty but with increasing rates in later life. The Triple-Negative Breast Cancer (TNBC) remains the most formidable subtype of breast cancer, accounting for approximately 15–20% of all cases globally. Unlike luminal subtypes, TNBC is defined by the absence of estrogen receptor (ER), progesterone receptor (PR), and human epidermal growth factor receptor 2 (HER2) expressions. However, its true challenge lies in its profound molecular heterogeneity; it is not a single disease entity but a complex cluster of subtypes, which is a primary driver of treatment failure and clinical relapse [2]. The aggressive clinical course of TNBC is characterized by high mitotic rates and a significant propensity for early visceral metastasis to the lungs and brain, with the risk of recurrence peaking within the first three years following diagnosis [3].

Historically, the mainstay of TNBC treatment was limited to cytotoxic chemotherapy (taxanes, anthracyclines, and platinum agents). While the therapeutic landscape has recently expanded to include targeted options such as immune checkpoint inhibitors (e.g., pembrolizumab) [4], PARP inhibitors for BRCA-mutated cases (e.g., Olaparib) [5], and antibody–drug conjugates (e.g., sacituzumab govitecan) [6], conventional chemotherapy remains the primary baseline treatment, particularly in resource-limited settings. While initially responsive a phenomenon known as the triple-negative paradox many patients develop multi-drug resistance (MDR) shortly after treatment initiation. This resistance is largely driven by the overexpression of ATP-binding cassette (ABC) transporters, such as P-glycoprotein (P-gp), which actively effluxes chemotherapeutic agents out of the tumour cells [7]. Recent evidence further suggests that the tumour microenvironment, particularly hypoxia, plays a critical role in up regulating these efflux pumps, thereby intensifying the chemo-resistant phenotype [8].

In Africa, TNBC is often diagnosed at advanced stages in younger women. The high cost of modern oncology drugs, coupled with limited access to radiotherapy, creates a severe clinical gap. Consequently, over 80% of the population relies on the continent’s rich biodiversity and endogenous medicinal plants as primary or complementary therapies. Africa’s diverse flora offers a massive but largely untapped library of bioactive secondary metabolites. However, the utilization of these genetic resources must be framed within the ethical guidelines of the Nagoya Protocol, ensuring the fair and equitable sharing of benefits arising from traditional botanical knowledge.

While various reviews have discussed medicinal plants for cancer in general, unlike broader reviews on medicinal plants and cancer, this review focuses specifically on African ethnomedicinal plants with reported activity in TNBC-relevant models. This review shifts the focus from general anticancer properties to the specific phytochemical richness of species such as *Phyllanthus muellerianus*, *Nauclea pobeguinii*, and *Xylopia aethiopica* [6]. By synthesizing ethnobotanical data with laboratory-verified IC_50_ values, this work aims to categorize these plants based on their traditional preparation methods ranging from aqueous decoctions to alcoholic infusions and their ability to inhibit the growth of aggressive cell lines like MDA-MB-231 associated with this aggressive type of cancer.

Ultimately, this review seeks to elevate the role of African biodiversity from traditional empirical use to a scientifically validated resource. By highlighting the potential for chemo-sensitization and therapeutic synergy with conventional drugs like doxorubicin, this work provides a roadmap for the development of standardized phytomedicines that are both accessible and effective in addressing the challenges of chemoresistance of this type of breast cancer.

## 2. Results

### 2.1. Perceptions of Cancer Within African Traditional Frameworks

Analysis of ethnobotanical reports indicates that indigenous communities throughout Southern, Western, and Central Africa hold a localized yet sophisticated perspective on oncological diseases. Although awareness is growing, a large segment of the population lacks a technical understanding of clinical oncology, relying instead on ancestral wisdom. A primary obstacle is the linguistic gap; many indigenous African dialects lack a precise terminology for cancer, leading the condition to be frequently identified by physical manifestations such as persistent ulcerations or abnormal internal growths rather than a singular medical diagnosis. This absence of a dedicated medical term often results in the disease being overlooked or misinterpreted within the local health discourse.

A diagnosis of a virulent malignancy, such as Triple-Negative Breast Cancer (TNBC), is widely perceived as an inescapable fatality. Cultural skepticism often surrounds Western medical interventions; many believe that surgical procedures or cytotoxic chemotherapy may disturb the tumor, causing it to metastasize or accelerating the patient’s decline [9]. Furthermore, the prohibitive costs and severe adverse effects associated with modern oncology drive a preference for phytotherapy. There is a prevailing cultural assumption that botanical remedies, particularly those derived from species like *Viscum album* offer a holistic and safer alternative to synthetic pharmaceuticals [10].

Dietary traditions also play a vital role in local cancer prevention strategies. In East and Southern Africa, the consumption of fresh extracts from Tulbaghia violacea or Curcuma longa is favored for their perceived purifying and detoxifying properties [11]. While communities often prioritize traditional oils and unprocessed pastes to avoid the carcinogens associated with refined foods, scientific literature warns that inadequate storage of these staples can result in contamination by mycotoxins, such as aflatoxins [9]. These fungal toxins are themselves highly carcinogenic, creating a paradoxical health risk. Ultimately, the African traditional concept of anti-TNBC care is characterized by a profound trust in the therapeutic potential of flora like Sutherlandia frutescens, highlighting a critical opportunity for the integration of traditional knowledge with modern pharmacological validation [12].

### 2.2. Ethnomedicinal Utilization of Anticancer Flora in African Communities

The field of cancer chemoprevention defined as the application of natural or synthetic substances to stall, obstruct, or reverse the process of carcinogenesis has seen a surge in scholarly attention across Africa [9]. These botanical agents serve as critical biological tools, acting either as blockers to prevent carcinogen exposure or as suppressors to halt the advancement of malignant tumors. Specifically, regarding Triple-Negative Breast Cancer (TNBC), numerous secondary metabolites extracted from African vegetation have exhibited the capacity to mitigate DNA damage and trigger cytotoxic responses in both cellular (*in vitro*) and animal (*in vivo*) models [13].

Our systematic investigation identified distinct plant species across 26 genera and 22 families with established traditional use or therapeutic potential against TNBC in Africa. Taxonomic analysis reveals that the most prominent families are Asteraceae (13%), followed by Fabaceae (7%), Annonaceae (7%), and Amaryllidaceae (7%). These families are recognized for their oncological applications not only in South, West, and East Africa but also globally. For example, *Catharanthus roseus*, indigenous to Madagascar and East Africa, is internationally renowned as the botanical origin of the chemotherapeutic agents vincristine and vinblastine [14].

**Figure 1:**
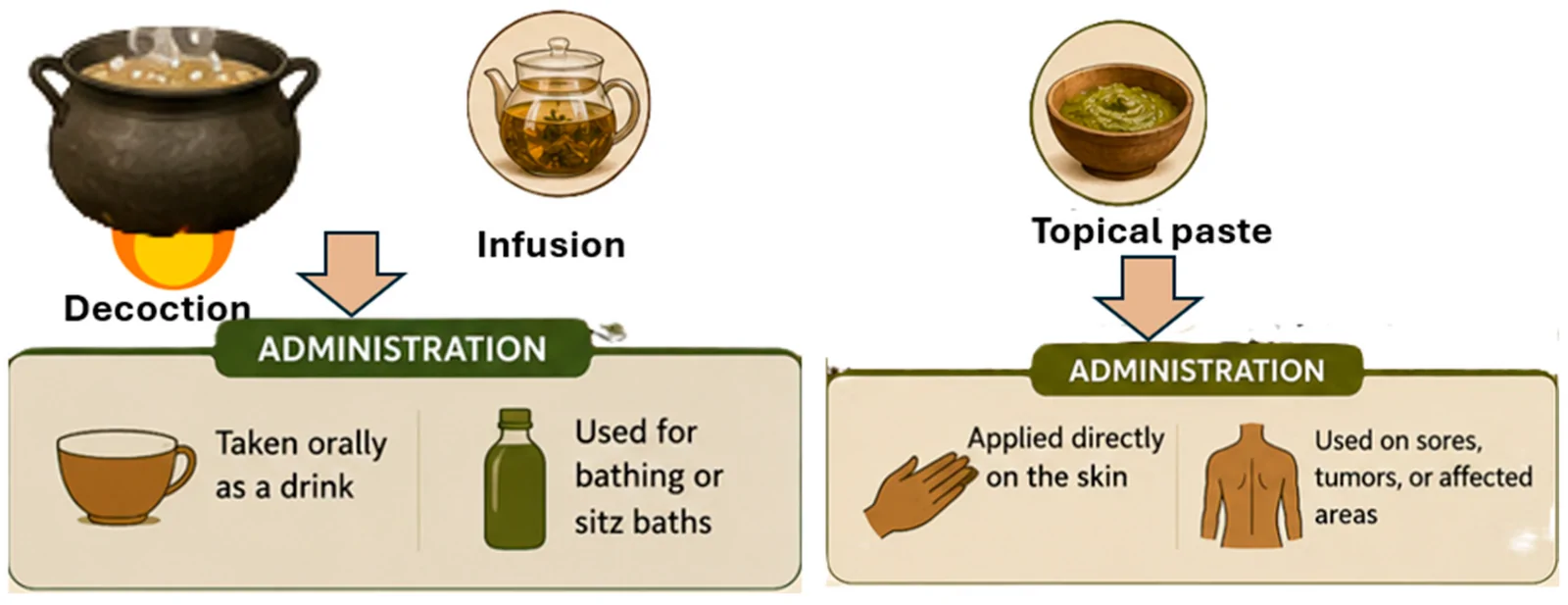
Different method of administration of TBNC traditional preparations.

Traditional African practitioners typically administer these remedies as decoctions, infusions, or topical pastes [13] (Table 1, Figure 1). Beyond purely medicinal use, several species serve as functional foods or spices; *Garcinia kola* and *Zanthoxylum zanthoxyloides* are often chewed for their bioactivity, while *Curcuma longa* and *Nigella sativa* are utilized in culinary practices for their chemopreventive properties [15]. Many of these plants are also integrated into complex polyherbal formulations. For instance, the seeds of *Peganum harmala* are utilized in aqueous infusions or inhaled as medicinal smoke for their rich alkaloid content [16] (Table 1).

### 2.3. Growth Habit, Parts Used, Preparation, and Mode of Administration

The botanical species identified in this review for their activity against Triple-Negative Breast Cancer (TNBC) are predominantly sourced from the wild, though a significant portion is cultivated for medicinal and dietary purposes (Table 1). These plants exhibit diverse growth habits, ranging from large trees like *Nauclea pobeguinii* to perennial herbs such as *Vernonia amygdalina* and *Tulbaghia violacea* with the remainder consisting of shrubs and climbing vines (Table 1 and Table 2).

Regarding the selection of botanical organs, the preparation of anti-TNBC extracts relies heavily on foliage and stem bark. Analysis of the identified flora shows that leaves (37%) are the most utilized part, seen in species like *Moringa oleifera* and *Artemisia annua*, followed by the use of bark (20%) (e.g., *Acacia erioloba*), roots (13%) (e.g., *Xylopia aethiopica*) and specialized storage organs such as bulbs and rhizomes (10%). The prevalence of leaves and bark in African traditional medicine is attributed to their regenerative capacity and high concentration of specialized secondary metabolites [90,91]. Furthermore, embryonal tissues and specialized storage organs, such as the bulbs of *Tulbaghia violacea* or the rhizomes of *Curcuma longa*, are also strategically employed for their rich phytochemical profiles [11,14].

In terms of galenic formulation, remedies are overwhelmingly administered as aqueous decoctions or infusions (57%) [92]. A substantial portion is also integrated directly into the diet as functional spices or consumed as raw masticatories (30%) [93], while a smaller fraction is processed into topical pastes for cutaneous application (13%) [90]. Plants are typically sourced from their natural habitats or home gardens according to immediate needs. Traditional practitioners often harvest specimens personally or collaborate with skilled collectors. Many maintain private botanical collections for highly demanded species, such as *Withania somnifera* or *Aloe ferox*, to ensure constant availability of fresh material [91,94]. Traditional pharmaceutical preparations are primarily documented as aqueous decoctions and infusions (Table 1). For example, the leaves of *Catharanthus roseus* and the bark of *Phyllanthus muellerianus* are typically boiled to facilitate the extraction of bioactive compounds. Beyond liquid extracts, several species are integrated into the daily diet as functional spices or consumed as masticatories, notably *Garcinia kola* and *Zanthoxylum zanthoxyloides* [13]. In cases involving external manifestations of the disease, plants are often crushed into a pulp to create topical ointments for direct application [90,93].

Harvesting occurs in natural ecosystems or local home gardens according to the immediate therapeutic need. Many practitioners maintain private botanical collections for rapid-growing species such as *Withania somnifera* to ensure the constant availability of biologically active fresh material. The administration of these remedies is overwhelmingly oral, although topical routes are preferred for malignancies that are accessible through the skin [90] (Table 1).

### 2.4. Other Ethnomedicinal Uses and Toxicity of the Reported Anti-TNBC Plants

Virtually all the botanical species catalogued in this systematic review are utilized within indigenous healthcare systems to address a broad spectrum of pathologies beyond oncology. A prominent example is *Moringa oleifera*, which, in addition to its anti-TNBC potential, is used for its anti-inflammatory, hypotensive, and antimicrobial properties across various African regions [95]. The fact that these plants are consistently integrated into community health practices for diverse therapeutic needs provides a compelling justification for their broad pharmacological efficacy and bioactive richness [96].

Conversely, several of the anticancer taxa identified in this study demonstrate significant toxicological profiles. A notable illustration is *Catharanthus roseus* (L.) G. Don. While it is a primary source of therapeutic agents like the indole alkaloids vincristine, these are documented for their potent neurotoxicity [97]. As potent antimitotic agents, vincristine and vinblastine exert their cytotoxic effects by binding to tubulin, which prevents the assembly of microtubules. This disruption inhibits the formation of the mitotic spindle, thereby halting mitosis during metaphase and leading to programmed cell death [98]. Consequently, clinical literature on these commercial derivatives highlights severe adverse reactions, including myelosuppression, peripheral neuropathy, alopecia, and gastrointestinal distress such as paralytic ileus and oral ulcerations [99]. *In vitro* evaluations of these pure alkaloids demonstrate high cytotoxic potency but a low selectivity index (SI) when compared against normal human cell models, underlining their narrow therapeutic window [99].

The inherent toxicity of these compounds necessitates rigorous dosage control to mitigate systemic adverse effects. This pharmacological reality partially clarifies why many traditional anti-TNBC preparations in Africa are either administered in minute quantities or applied as topical poultices. Interestingly, the localized application of fresh extracts or ointments as seen with species like *Tulbaghia violacea* serves as a strategic method to target external tumors directly while minimizing systemic toxicity and reducing the risk of internal organ damage [11]. Similarly, while raw extracts of deeply characterized plants like *Vernonia amygdalina* and *Curcuma longa* show relative safety in traditional multi-target uses [11], the sparse availability of direct IC_50_ ratios against normal mammary lines (e.g., MCF-10A) remains a parameter that restricts the formal mathematical calculation of their selectivity indices within the current primary literature [83,100].

### 2.5. Methodological Quality and Risk of Bias Assessment

The methodological quality screening of the 52 included primary in vitro studies revealed a diverse distribution in reporting rigor across the six evaluated domains. Applying the tailored 12-point scoring matrix, the majority of the analyzed literature demonstrated moderate to high quality (score > 6). High compliance was generally observed in cell line specifications and extraction protocols; however, critical gaps were identified regarding formal botanical voucher deposition formal cell-line authentication, selectivity indexes on non-cancerous cells, and the systematic inclusion of standardized chemotherapy positive controls (Table 3). This variation highlights the crucial need for standardized reporting frameworks in ethnopharmacological research to minimize experimental bias.

### 2.6. Categorization of Mechanistic Evidence Levels

To objectively evaluate the pharmacological relevance and specificity of the included botanical species toward triple-negative breast cancer, literature data were stratified into five distinct, hierarchical levels of evidence based on the experimental models utilized: Level 1 (Direct TNBC Evidence) involving TNBC-specific cell lines (e.g., MDA-MB-231, 4T1); Level 2 (General Breast Cancer Evidence) demonstrating activity in non-TNBC breast cancer models (e.g., MCF-7, T47D); Level 3 (Non-Breast Malignancy Evidence) documenting cytotoxicity strictly limited to other cancer models (e.g., leukemia, colon, lung); Level 4 (Computational Evidence) restricted solely to *in silico* profiling or molecular docking without experimental validation; and Level 5 (Ethnobotanical Evidence) based exclusively on traditional healthcare documentation (Table 1 and Table 2). This classification framework maps the current research status of each species along this translational hierarchy, effectively accounting for cases within this systematic review where direct anti-TNBC mechanistic data remain currently unavailable, such as for *Phyllanthus muellerianus*, *Maclura pomifera*, *Myrothamnus flabellifolius*, *Vachellia erioloba* (syn. *Acacia erioloba*), *Nauclea pobeguinii*, *Xylopia aethiopica*, *Crinum zeylanicum*, and *Bupleurum rotundifolium*.

### 2.7. Metabolites That May Be Contributing to the Pharmacological Activity of These Plants

Rigorous scientific analysis has isolated several potent cytotoxic constituents from these plants. Noteworthy examples include Artemisinin from *Artemisia annua*, which utilizes an endoperoxide moiety to trigger ferroptosis and oxidative stress in malignant cells [52,53,54]. Furthermore, Withaferin A derived from *Withania somnifera* has shown exceptional efficacy against the MDA-MB-231 and BT-549 TNBC cell lines [50,51,101,102]. Other significant compounds include the cytotoxic sesquiterpene lactone Vernodalin from *Vernonia amygdalina* [103,104,105]. These phytochemicals are effective because they engage diverse molecular targets, resulting in apoptosis induction, cell cycle arrest, and the inhibition of tumor angiogenesis. The synergy between antioxidant capacity and cytotoxic action provides a comprehensive pharmacological strategy for addressing aggressive malignancies like TNBC [53].

### 2.8. Anti-TNBC Potential of Compounds from African Plants

#### 2.8.1. Triterpenoids and Steroids

These classes represent the most promising group in our study, encompassing molecules with diverse mechanisms of action. Withaferin A stands out with exceptional efficacy, showing IC_50_ values between 0.5 and 2.5 µM across MDA-MB-231, BT-549, and SUM159 lines. Nimbolide, epoxyazadiradione, and gedunin, derived from *Azadirachta indica*, also show strong cytotoxicity, with nimbolide being the most potent (2–12 µM) [43,44,84]. Saikosaponin D (10.2 µM) and alpha-hederin (10 µM) exhibit significant activities on MDA-MB-231 [74,75,87]. Other compounds, such as asiatic acid and asiaticoside, show variable results depending on the study, while sutherlandiosides A–D are noted for their synergistic effects [24,72].

#### 2.8.2. Flavonoids and Glycosides

Flavonoids primarily act by modulating cell signaling cascades. Chrysosplenol D is the leader of this category, with an IC_50_ of 22.1 µM against MDA-MB-231 and BT-20, acting through the ERK1/2 pathway [52,53,54]. Silibinin, although presenting lower direct cytotoxicity (50–200 µM), is crucial for inhibiting the motility and invasion of MDA-MB-231 and Hs578T cells. Quercetin shows notable activity at 20 µM, unlike its glycoside, rutin (3), which is much less active (137–143 µM) [23,85,95].

#### 2.8.3. Alkaloids and Sesquiterpene Lactones

Alkaloids such as harmine and harmaline demonstrate significant cytotoxicity, particularly harmine (<25 µM), and their efficacy is enhanced when combined with nedaplatin. Dihydrochelerythrine specifically targets the BT-549 line with an IC_50_ of 21.2 µM [106,107]. In the sesquiterpene lactone group, deoxyelephantopin and isodeoxyelephantopin are extremely high performers against MDA-MB-231 and BT-549, with values around 9.5–11.2 µM [76,77]. Artemisinin, while a pillar of antimalarial treatment, shows more discrete and variable anticancer activity (from 25 µM to >100 µM) on TNBC strains [52,53,54].

#### 2.8.4. Phenols, Quinones, and Other Compounds

**Figure 2:**
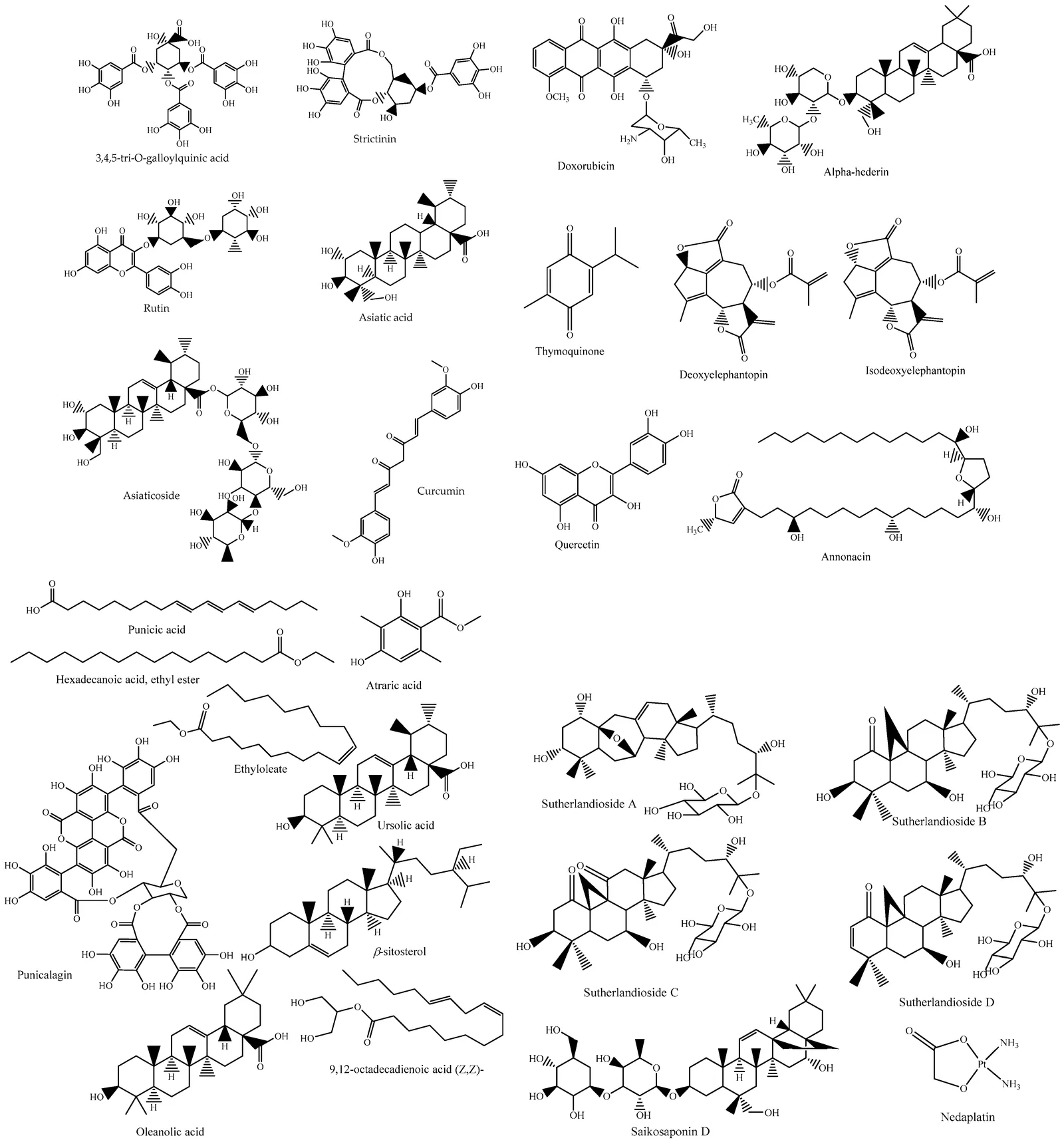

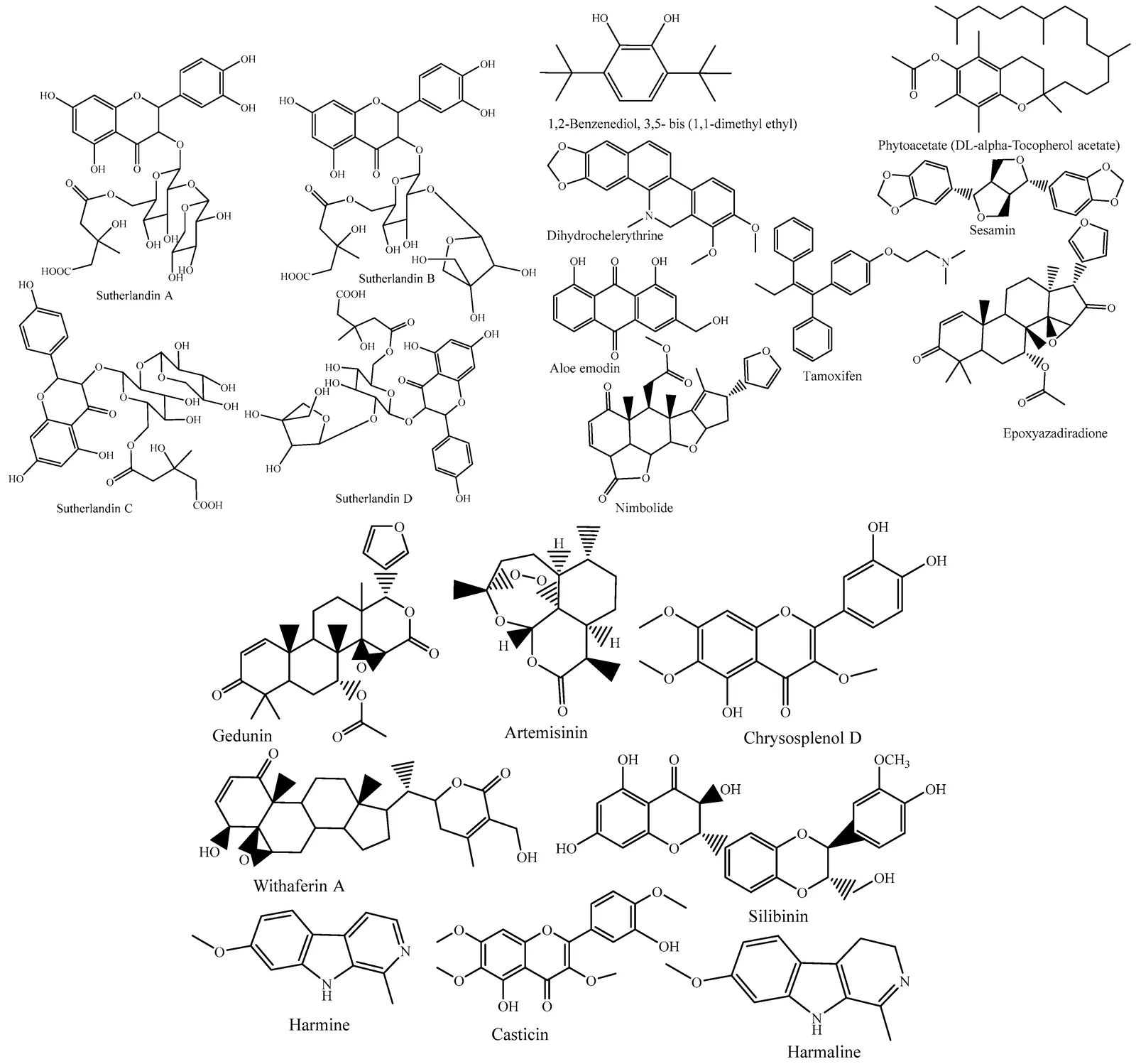
Some of the anti-TNBC molecules reported in African plants used in Traditional medicine to manage TNBC. They correspond to the molecules mentioned in Table 2.

Thymoquinone, extracted from *Nigella sativa*, is one of the most active phenolic compounds (5–20 µM against MDA-MB-231). Curcumin displays versatile activity on MDA-MB-231, MDA-MB-468, and the murine 4T1 line, although its IC_50_ values vary from 15 to 50 µM depending on the study [73,74,75]. Annonacin proves to be very powerful, particularly on MDA-MB-468 (8.5 µM) [79]. Finally, other molecules, such as strictinin and 3,4,5-tri-O-galloylquinic acid, show stable activity around 30 µg/mL [21,22,71]. Reference agents like doxorubicin and tamoxifen serve as benchmarks to evaluate the potency of these natural candidates Figure 2.

### 2.9. Mechanistic Insights and Phytochemical-Based Therapeutics of African Medicinal Plants Targeting Triple-Negative Breast Cancer

Rigorous scientific analysis has identified and isolated several potent cytotoxic constituents from medicinal plants from Africa (Table 2, Figure 2). Extracts of these plants and their chemical constituents are documented to exhibit exceptional cytotoxic potency against TNBC strains (Table 1, Table 2). The most potent agents identified highlight the diversity of high-potency secondary metabolites including alkaloids, terpenoids and lectins that target aggressive TNBC cell lines with precision. These phytochemicals are effective because they engage diverse molecular targets, resulting in apoptosis induction, cell cycle arrest, and the inhibition of tumor angiogenesis. The exceptional efficacy of these phytochemicals in TNBC stems from their ability to modulate multiple signaling pathways simultaneously, beyond simple cytotoxicity, by engaging a multi-pronged attack on cancer cell survival. These compounds trigger the intrinsic apoptotic pathway and disrupt pro-survival axes, such as the EGFR/PI3K/Akt/mTOR signaling pathway targeted by metabolites such as withaferin A and silibinin [85,102], or the STAT3 phosphorylation blocked by isodeoxyelephantopin [76,77].

*Tulbaghia violacea*

*Tulbaghia violacea* has emerged as a promising African medicinal plant with potential activity against triple-negative breast cancer (TNBC) due to its diverse phytochemicals that modulate oxidative stress, inflammatory signalling, apoptosis, and cell-cycle regulation. Studies using the TNBC cell line MDA-MB-231 show that extracts of *T. violacea* induce oxidative stress-mediated mitochondrial apoptosis. Increased intracellular reactive oxygen species (ROS) disrupt mitochondrial membrane potential, leading to activation of pro-apoptotic Bax and suppression of anti-apoptotic Bcl-2. This promotes mitochondrial outer membrane permeabilization and release of cytochrome-c, which activates caspase-9 and downstream caspase-3, resulting in PARP cleavage, DNA fragmentation, and apoptotic cell death. Persistent oxidative stress also contributes to DNA damage and replication stress, further reducing TNBC cell viability [11,14,17,18].

Another important mechanism involves inhibition of the NF-κB signaling pathway, a key regulator of TNBC survival and metastasis. Bioactive constituents of *T. violacea* suppress NF-κB activation and nuclear translocation, thereby downregulating anti-apoptotic proteins such as Bcl-2 and survivin and reducing inflammatory cytokine signalling. This inhibition weakens tumor-promoting inflammatory pathways and enhances apoptotic responses. *T. violacea* extracts also disrupt cell-cycle progression, causing accumulation of cells in the S-phase and a reduction in the G0/G1 phase. This shift indicates replication stress and activation of S-phase checkpoints that inhibit DNA replication and cell proliferation. In addition, the extract may modulate inflammatory enzymes such as Cyclooxygenase-2, whose overexpression in breast tumors promotes prostaglandin-mediated inflammation, angiogenesis, and tumor progression. Inhibition of COX-2 reduces prostaglandin synthesis and contributes to suppression of tumor growth [11,14,17,18]. Several phytochemicals identified in *T. violacea* are associated with these effects, including 2,3-dihydro-3,5-dihydroxy-6-methyl-4H-pyran-4-one (DDMP), α-Pinene, Cuminaldehyde, and Pyrrolidine derivatives. DDMP contributes to NF-κB suppression and oxidative stress modulation, α-pinene induces cell-cycle arrest through regulation of cyclin-dependent kinases, cuminaldehyde triggers mitochondrial apoptosis via loss of membrane potential and caspase activation, while pyrrolidine derivatives promote intrinsic and extrinsic apoptotic signaling. Collectively, these mechanisms highlight the multi-target anti-TNBC potential of *Tulbaghia violacea* [11,14,17,18].

*Catharanthus roseus*

*Catharanthus roseus* is renowned for producing terpenoid indole alkaloids, especially vincristine and vinblastine, which are among the most established plant-derived anticancer agents [98,99,108,109]. These vinca alkaloids exert their anticancer activity by binding to tubulin, the building block of microtubules, thereby disrupting microtubule dynamics critical for mitotic spindle formation during cell division. This disruption halts the cell cycle at the G2/M phase, preventing chromosome segregation and leading to mitotic arrest and apoptotic cell death in proliferating cancer cells, including models of breast cancer. Mechanistically, vincristine and vinblastine bind to tubulin heterodimers, inhibiting polymerization and destabilizing microtubule structures. This interruption of cytoskeletal dynamics activates cell-cycle checkpoints, triggers caspase-mediated apoptosis, and enhances the intrinsic apoptotic pathways in cancer cells. Although classic use of these alkaloids is in chemotherapeutic regimens rather than crude plant extracts, their mechanisms of mitotic inhibition and apoptosis induction are directly relevant to TNBC cell models (e.g., MDA-MB-231), where agents that disrupt cell division and trigger apoptotic signaling have clinical utility [19,20,108,109].

*Phyllanthus muellerianus*

While extensive mechanistic data remains limited, preliminary evidence explicitly evaluates *Phyllanthus muellerianus* in breast cancer models. Specifically, methanolic extracts of its leaves exert significant cytotoxic activity *in vitro* against breast cancer cell lines, including the triple-negative breast cancer model MDA-MB-231. The broader anticancer mechanisms of the plant are further supported by studies demonstrating anti-proliferative and pro-apoptotic effects in human prostate cancer PC-3 cells via the activation of executioner caspases (caspases 3/9) and modulation of intracellular calcium influx. These findings align with the broader *Phyllanthus* genus profile, where polyphenolic compounds frequently inhibit cell proliferation, migration, and invasion while successfully triggering apoptosis [65,110].

*Melia azedarach*

Preliminary mechanistic data demonstrates that *Melia azedarach* extracts possess selective anticancer activities across multiple human breast cancer models, including the triple-negative breast cancer line MDA-MB-231 and hormone-receptor-positive T47D cells. Phytochemical screenings and fractionation analyses of its leaves, fruits, and bark reveal an enrichment in bioactive compounds such as limonoids, triterpenoids, steroids, and flavonoids. These classes contribute directly to significant in vitro cytotoxicity, antiproliferative effects, and the inhibition of lysosomal acidification. Furthermore, experimental evidence from animal models indicates that combination treatments involving *M. azedarach* extracts effectively suppress tumor growth in vivo while shifting cellular balance toward programmed cell death, notably through the upregulation of pro-apoptotic markers like Bax and the induction of cell cycle arrest [84,111,112,113,114,115,116,117].

*Maclura pomifera*

*Maclura pomifera* extracts, particularly those containing pomiferin and osajin, exhibit anti-proliferative and pro-apoptotic effects in breast cancer models via the activation of TRPV1 channels [118,119]. Additionally, semi-synthetic derivatives of these isoflavones target G-quadruplex DNA to induce cell cycle arrest, providing a mechanism for potential anti-metastatic actions [120].

*Myrothamnus flabellifolius*

Experimental evidence explicitly demonstrates that *Myrothamnus flabellifolius* possesses potent and selective anti-TNBC activity. Phytochemical evaluations have led to the isolation of strictinin, an ellagitannin from this species that exhibits pronounced cytotoxicity against TNBC cell lines (such as MDA-MB-231 and BT-549) with minimal effects on non-malignant breast epithelial cells [21,22,71]. Mechanistically, strictinin acts as a novel ROR1 inhibitor, successfully repressing TNBC survival and metastatic migration by modulating the PI3K/AKT/GSK3β signaling pathway [71]. Furthermore, crude extracts from the plant have been shown to alter oncogenic microRNA profiles, effectively restoring estrogen receptor alpha (ERα) expression and re-sensitizing TNBC cells to hormone therapies like tamoxifen [22]. These targeted pathways contrast with the plant’s broader, non-specific apoptotic potential observed in other malignancies, such as myeloid leukemia systems [121].

*Vachellia erioloba*

Direct mechanistic studies evaluating *Vachellia erioloba* extracts specifically within triple-negative breast cancer models remain restricted. However, the broader pharmacological profile of this species demonstrates clear anticancer and medicinal potential. Recent investigations highlight that bio-resource extracts from *V. erioloba* seeds can be successfully utilized for the green synthesis of zinc oxide nanoparticles, which exhibit significant in vitro cytotoxic and antiproliferative activities against human cancer lines through apoptosis induction. Additionally, organic extracts from the plant possess verified anti-inflammatory properties, characterized by the targeted inhibition of the 15-lipoxygenase (15-LOX) enzyme and the suppression of nitric oxide release in macrophage models [23,122].

*Nauclea pobeguinii*

*Nauclea pobeguinii* extracts exhibit profound in vitro cytotoxic and antiproliferative activities against a wide panel of human malignancies, spanning drug-sensitive genotypes as well as multi-factorial drug-resistant cancer cell lines. Investigations utilizing the triple-negative breast cancer model MDA-MB-231 (including its breast cancer resistance protein-overexpressing subline, MDA-MB-231-BCRP) demonstrate that methanolic bark extracts from this species induce significant collateral sensitivity. Phytochemical fractionation of these active extracts has led to the characterization of specific bioactive constituents, establishing stilbenes predominantly resveratrol and resveratrol *β*-D-glucopyranoside alongside indole alkaloids like strictosamide, as the key drivers inducing growth inhibition and apoptosis across both breast and colorectal carcinoma frameworks [29,30,123].

*Xylopia aethiopica*

Recent original research provides direct mechanistic evidence of the efficacy of *Xylopia aethiopica* against triple-negative breast cancer (TNBC) models. In vitro evaluation of its fruit and root solvent fractions reveals potent antiproliferative activity against highly aggressive HCC-70 and MDA-MB-231 cell lines, with an IC_50_ value reaching as low as 19.96 µg/mL [35]. Furthermore, 13C-NMR metabolic profiling and molecular docking studies have identified core bioactive constituents, notably kaurane-type diterpenoids and related alkaloids, capable of binding to target oncogenic proteins to trigger caspase-dependent apoptosis and suppress tumor progression [31,32,78].

*Sutherlandia frutescens*

*Sutherlandia frutescens* exhibits antiproliferative and pro-apoptotic effects in various breast cancer models. Extracts and compounds such as sutherlandiosides and sutherlandins induce reactive oxygen species (ROS) production, disrupt mitochondrial membrane potential, increase Bax/Bcl-2 ratio, and activate caspase-3, leading to apoptosis. Additionally, *S. frutescens* extracts inhibit NF-κB signaling, reduce expression of pro-inflammatory cytokines, and block migration and invasion by downregulating MMPs mechanisms highly relevant to TNBC aggressiveness [39,82].

*Hypoxis hemerocallidea*

Also known as African potato, *Hypoxis hemerocallidea* has limited direct TNBC mechanistic data. Some studies with extracts indicate antioxidant activity and modest cytotoxicity in breast cancer cells. The presence of sterols and norlignans suggests potential pro-apoptotic and anti-proliferative effects, but robust mechanistic evidence in TNBC cell lines has not been published [66,67,88,124].

*Vernonia amygdalina*

*Vernonia amygdalina* (bitter leaf) has documented antitumor effects in breast cancer models, including TNBC cell lines [125,126]. Mechanistic studies indicate that extracts induce cell-cycle arrest (G0/G1 and S-phase), increase ROS, and trigger mitochondrial apoptosis through upregulation of Bax, activation of caspase-3, and PARP cleavage. Vernodalin and related sesquiterpene lactones isolated from *V. amygdalina* exhibit significant cytotoxicity and modulate signalling pathways such as PI3K/Akt and NF-κB, contributing to reduced proliferation and increased apoptosis in TNBC cells [126].

*Garcinia kola*

Extracts of *Garcinia kola* (bitter kola) seeds show cytotoxic activity in breast cancer cells, and the major constituent kolaviron a biflavonoid complex induces apoptosis and inhibits proliferation. Mechanistically, kolaviron increases ROS, modulates Bax/Bcl-2 balance, activates caspase-3, and suppresses NF-κB signalling. These effects reduce survival signalling and promote apoptotic death in cancer cells, including models relevant to TNBC [68,69,127].

*Zanthoxylum zanthoxyloides*

There is limited mechanistic data on anti-TNBC activity of *Zanthoxylum zanthoxyloides*. Extracts have demonstrated general antiproliferative and cytotoxic effects in cancer models, potentially via apoptosis induction and modulation of oxidative stress, but no specific TNBC mechanisms or isolated active compounds have been conclusively reported [128].

*Aloe ferox*

*Aloe ferox* extracts and constituents such as aloin and aloe-emodin demonstrate antiproliferative and pro-apoptotic effects in breast cancer cells. Mechanistically, these compounds increase ROS, trigger mitochondrial apoptosis, inhibit NF-κB signalling, and induce cell-cycle arrest. While specific TNBC cell line studies are limited, the pathways modulated (ROS, apoptosis, NF-κB) are directly relevant to TNBC pathobiology [70,89,129].

*Curcuma longa*

Curcumin, the principal curcuminoid from *Curcuma longa* (turmeric), has well-characterized anticancer mechanisms in TNBC. Curcumin induces ROS, disrupts mitochondrial membrane potential, and triggers apoptosis via activation of caspase-3 and caspase-9. It also inhibits NF-κB signaling, suppresses STAT3 and PI3K/Akt pathways, and induces cell-cycle arrest (G2/M), collectively reducing proliferation, survival, and metastatic potential in TNBC cells [26,73,130].

*Annona muricata*

*Annona muricata* (graviola) contains annonaceous acetogenins such as annonacin that potently inhibit cancer cell proliferation [79]. Mechanisms involve mitochondrial complex I inhibition, leading to decreased ATP production, increased ROS, loss of mitochondrial membrane potential, and induction of apoptosis. These compounds also trigger cell-cycle arrest and suppress survival signalling pathways such as NF-κB, making them mechanistically relevant to TNBC inhibition where metabolic vulnerability and apoptotic evasion are critical [33,34,35,36,37]. Annonacin modulate PD-L1 and interferon-gamma expression to attenuate cancerous features [37].

*Moringa oleifera*

*Moringa oleifera* leaf and seed extracts exhibit antiproliferative, pro-apoptotic, and anti-inflammatory activity in breast cancer models [58,59,60,61,95]. Mechanistically, Moringa compounds such as isothiocyanates and flavonoids increase ROS, modulate Bax/Bcl-2 ratios, activate caspases, and suppress PI3K/Akt signaling. These pathways reduce cell viability and enhance apoptosis, and evidence from TNBC cell studies supports cell-cycle arrest and inhibition of migration and invasion [58,59,60,61,131]. The extracts from *Moringa oleifera* and *Azadirachta indica* target the VEGF pathways essential for new blood vessel formation [43,44,58,59]. The synergy between antioxidant capacity and cytotoxic action provides a comprehensive pharmacological strategy for addressing aggressive malignancies like TNBC [95].

*Nigella sativa*

The major bioactive constituent thymoquinone from *Nigella sativa* seeds exhibits pronounced activity in TNBC models. *Nigella sativa* containing thymoquinone and alpha-hederin from show strong activity at 5–20 µM and 10 µM, respectively [27,74,75]. Other notable molecules with significant micromolar potency include isodeoxyelephantopin from *Elephantopus scaber* (IC_50_ ~9.5 µM) [77]. Mechanistically, thymoquinone increases ROS, induces mitochondrial apoptosis via Bax upregulation and Bcl-2 downregulation, activates caspases, and inhibits NF-κB and STAT3 signaling. It also induces cell-cycle arrest and suppresses metastatic markers, illustrating multi-target engagement in TNBC cells [132,133,134].

*Punica granatum*

*Punica granatum* (pomegranate) extracts and ellagitannins such as punicalagin exhibit anti-TNBC activity by inducing ROS, initiating mitochondrial apoptosis, activating caspases, and suppressing NF-κB and PI3K/Akt signaling. These mechanisms lead to reduced proliferation, increased apoptosis, and inhibition of migration and invasion in TNBC cell lines [38,80,81].

*Centella asiatica*

*Centella asiatica* (Gotu kola) has demonstrated anticancer activity in breast cancer models, though specific anti-TNBC mechanisms are limited in the literature. Extracts and compounds such as asiaticoside, madecassoside, asiatic acid, and madecassic acid modulate apoptotic pathways, increase ROS, and inhibit proliferative signaling. Mechanistically, asiatic acid induces mitochondrial apoptosis via upregulating Bax, downregulating Bcl-2, and activating caspases in breast cancer cells. Additionally, *C. asiatica* extracts can suppress NF-κB signaling and reduce angiogenic mediators such as VEGF, implying anti-proliferative and anti-metastatic effects. Because TNBC is characterized by aggressive proliferation and metastatic potential, these mechanisms are highly relevant even though direct studies in TNBC cell lines (e.g., MDA-MB-231) are not yet widely documented [24,72].

*Elephantopus scaber*

Original laboratory research studies provide direct mechanistic evidence of the anti-TNBC efficacy of *Elephantopus scaber* active constituents. *In vitro* screening demonstrates that its primary isolated germacranolide sesquiterpene lactones, such as isodeoxyelephantopin, potently induce cell death and inhibit proliferation in human MDA-MB-231 triple-negative breast cancer networks. Mechanistically, these compounds force apoptotic collapse and mitochondrial stress by selectively blocking STAT3 phosphorylation pathways, downregulating survival signals, and significantly enhancing the therapeutic sensitivity of TNBC cell systems to standard chemotherapeutic agents like paclitaxel [28,76,77,135].

*Crinum zeylanicum*

Empirical in vitro investigations offer direct, level 1 mechanistic proof validating the antineoplastic potential of *Crinum zeylanicum* within triple-negative breast cancer (TNBC) systems. Through targeted bio-guided fractionation, researchers successfully isolated key Amaryllidaceae isoquinoline alkaloids specifically lycorine, ambelline, and crinine from the plant matrix. When deployed against highly invasive human MDA-MB-231 cell lines, pure crinine drives a severe, concentration-dependent suppression of tumor proliferation by triggering systematic apoptotic death, marked by hallmark nuclear fragmentation and structural disintegration. Furthermore, structure-activity relationship (SAR) profiling reveals that the preservation of an unreduced ∆^1,2^ double bond in the core crinane architecture is biochemically indispensable for driving this targeted cytotoxic response [25,136].

*Silybum marianum*

*Silybum marianum* (milk thistle) is rich in flavonolignans, collectively called silymarin; the most studied is silibinin. Silibinin has extensive anticancer evidence and has been tested in TNBC cell lines. Mechanistically, silibinin induces mitochondrial apoptotic pathways by elevating Bax and caspase-3 activity while attenuating Bcl-2. It also inhibits STAT3, NF-κB, and PI3K/Akt signalling, reduces cell proliferation, and induces G0/G1 cell-cycle arrest. In TNBC models specifically, silibinin has been shown to attenuate stemness features and inhibit migratory/invasive behavior by targeting epithelial–mesenchymal transition (EMT) regulators [137]. To combat the highly metastatic nature of TNBC, these secondary metabolites effectively inhibit tumor angiogenesis and migration; for instance, silibinin suppresses TGF-*β*2 expression to inhibit cell motility, isodeoxyelephantopin downregulates MMP-2/9 to retard migration [77,135].

*Viscum album*

*Viscum album* (European mistletoe) extracts exert anti-cancer effects primarily through lectins and viscotoxins. Mistletoe lctins (ML-I), reach nanogram-level efficacy with an IC_50_ of approximately 4.2 ng/mL in 2D models and 12.8 ng/mL in 3D models against MDA-MB-231 [48,49]. In breast cancer models (including TNBC), mistletoe lectins trigger apoptosis via mitochondrial pathways, increase ROS, activate caspases, and induce immunogenic cell death. Viscotoxins can disrupt membrane integrity and augment apoptotic signaling. These extracts also modulate immune responses by activating NK cells and increasing cytokine release, which may support tumor cell recognition and clearance. While *V. album*’s actions are less mechanistically specific than small-molecule phytochemicals, the combined pro-apoptotic, ROS-mediated, and immune-modulatory effects are relevant to TNBC pathobiology [48,49].

*Withania somnifera*

*Withania somnifera* (ashwagandha) produces withanolides such as withaferin A that have been evaluated in TNBC cell lines. Withaferin A derived has shown exceptional efficacy against the MDA-MB-231 (IC_50_ ~0.5–2.0 µM) and BT-549 (IC_50_ ~1.5 µM) TNBC cell lines [50,51]. Withaferin A induces ROS-mediated apoptosis, triggers mitochondrial dysfunction, and activates caspase cascades. It also inhibits NF-κB, STAT3, and Akt pathways, leading to decreased proliferation and survival signaling. Withaferin A reduces vimentin expression and EMT, impairing migratory and invasive capacities, and induces G2/M cell-cycle arrest [101,138,139].

*Artemisia annua*

*Artemisia annua* produces artemisinin and related sesquiterpene lactones. In TNBC models, artemisinin derivatives induce ROS-mediated mitochondrial apoptosis, increase Bax, reduce Bcl-2, and activate caspase-3. Artemisinin also inhibits Wnt/β-catenin and NF-κB pathways, suppressing proliferation, migration, and invasion. Additionally, these compounds can generate iron-dependent free radicals selectively in cancer cells, enhancing cytotoxic specificity [52,53,54,140]. Noteworthy artemisinin utilizes an endoperoxide moiety to trigger ferroptosis and oxidative stress in malignant cells [53]. The flavonoid chrysosplenol D from this plant, displays an IC_50_ of 22.1 µM against MDA-MB-231. The anticancer mechanism of this phytochemical involves the modulation of signaling cascades; inducing apoptosis in TNBC cells specifically through an ERK1/2-mediated pathway [52,53,54].

*Bupleurum rotundifolium*

While direct mechanistic investigations on triple-negative breast cancer systems are not yet available for raw *Bupleurum rotundifolium* extracts, the plant possesses distinct antiproliferative and oncological potential [55]. Phytochemical evaluations of its fruits have led to the isolation of unique triterpenoid glycosides, specifically named rotundifoliosides A-G, which exhibit marked cytotoxicity against human tumor cell lines. Furthermore, nanomedicinal applications utilizing *B. rotundifolium* extracts for the green synthesis of silver nanoparticles (AgNPs) demonstrate significant, dose-dependent biological and anticancer activities, reflecting the broader therapeutic efficacy observed within the *Bupleurum* genus [55,87].

*Peganum harmala*

*Peganum harmala* seeds contain *β*-carboline alkaloids (e.g., harmine, harmaline, norharmane) with known anticancer actions. These alkaloids intercalate DNA and inhibit topoisomerases, leading to cell-cycle arrest and apoptosis. They also generate ROS and modulate MAPK and Akt signalling in breast cancer cells. Mechanistic evidence in TNBC models specifically is limited, but the pathways impacted DNA damage, apoptosis, oxidative stress, and survival signalling inhibition align with vulnerabilities in TNBC biology [141,142].

*Azadirachta indica*

Cumulative data reviewed in the literature emphasizes the significant therapeutic value of *Azadirachta indica* extracts and its isolated compounds against highly aggressive malignancies, including triple-negative breast cancer systems. Specifically, methanolic and ethanolic extracts obtained from the leaves, flowers, bark, and seeds exhibit direct, dose-dependent cytotoxicity in breast carcinoma cell lines, with crude fraction thresholds documented near 62.5 µg/mL. Furthermore, nimbolide, a major active tetranortriterpenoid limonoid isolated from this species, demonstrates enhanced efficacy, displaying lower inhibitory concentration values ranging from 2 µM to 12 µM. Mechanistically, these agents effectively disrupt cell proliferation, impair metastatic invasion, and trigger caspase-dependent apoptotic pathways in malignant models [43,44].

**Figure 3:**
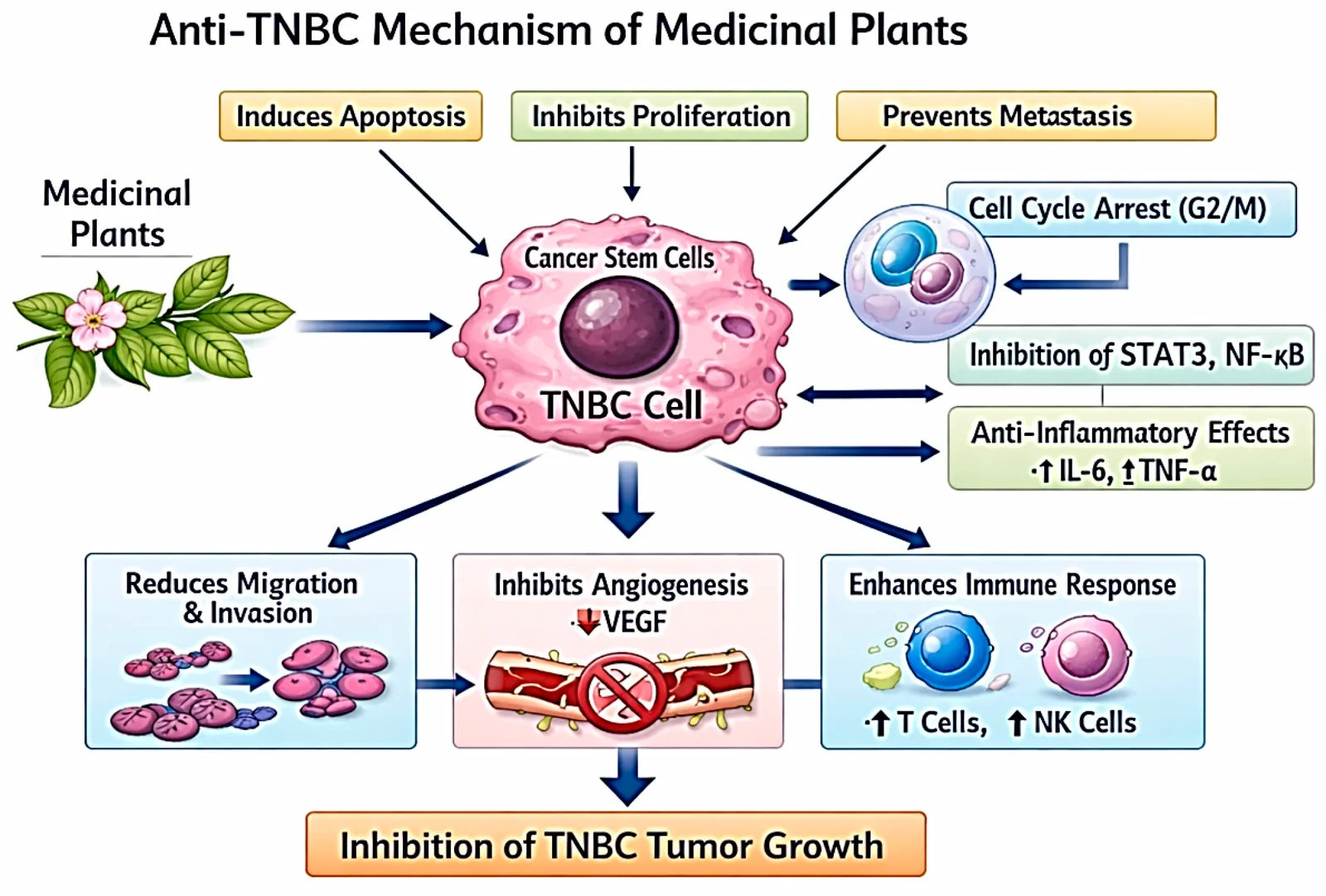
Schematic Representation of the Anti-TNBC Mechanisms of Medicinal Plants.

Figure 3 illustrates the multi-targeted anticancer mechanisms by which medicinal plants exert inhibitory effects against Triple Negative Breast Cancer (TNBC). Medicinal plants, represented as the primary therapeutic source, contain diverse bioactive constituents that interact with cancer cells and modulate several molecular and cellular pathways involved in tumor progression. Unlike single-target conventional therapies, these plant-derived compounds act on multiple biological processes simultaneously, thereby enhancing their therapeutic potential against aggressive cancers such as TNBC [101,102]. Recent integrated chemical and biological characterizations of complex plant matrixes, such as those performed on *Hypericum perforatum*, further support this paradigm by demonstrating how synergistically profiled extracts concurrently strike multiple functional in vitro oncological targets [143].

At the core of the diagram is the TNBC cell, which serves as the principal target of these bioactive compounds. Arrows originating from medicinal plants indicate three fundamental anticancer actions: induction of apoptosis, inhibition of proliferation, and prevention of metastasis. The induction of apoptosis represents a critical mechanism whereby medicinal plants activate programmed cell death pathways in TNBC cells. This process typically involves mitochondrial dysfunction, activation of caspases, and regulation of pro- and anti-apoptotic proteins, ultimately leading to the selective elimination of malignant cells without affecting normal tissues [105,114]. This multi-pronged activation of the apoptotic core has been recently validated through comparative extractions of traditional taxa like *Urtica dioica*, where specific solvent fractions directly trigger caspase-dependent apoptotic pathways and regulate cell survival biomarkers [144].

In addition to promoting apoptosis, medicinal plants inhibit the proliferation of TNBC cells. This is achieved through interference with the cell cycle machinery, often resulting in cell cycle arrest at key checkpoints such as the G2/M phase. By disrupting the orderly progression of the cell cycle, these compounds effectively reduce the rate of cancer cell division and limit tumor expansion. This antiproliferative effect is essential in controlling the rapid and uncontrolled growth characteristic of TNBC.

**Figure 4:**
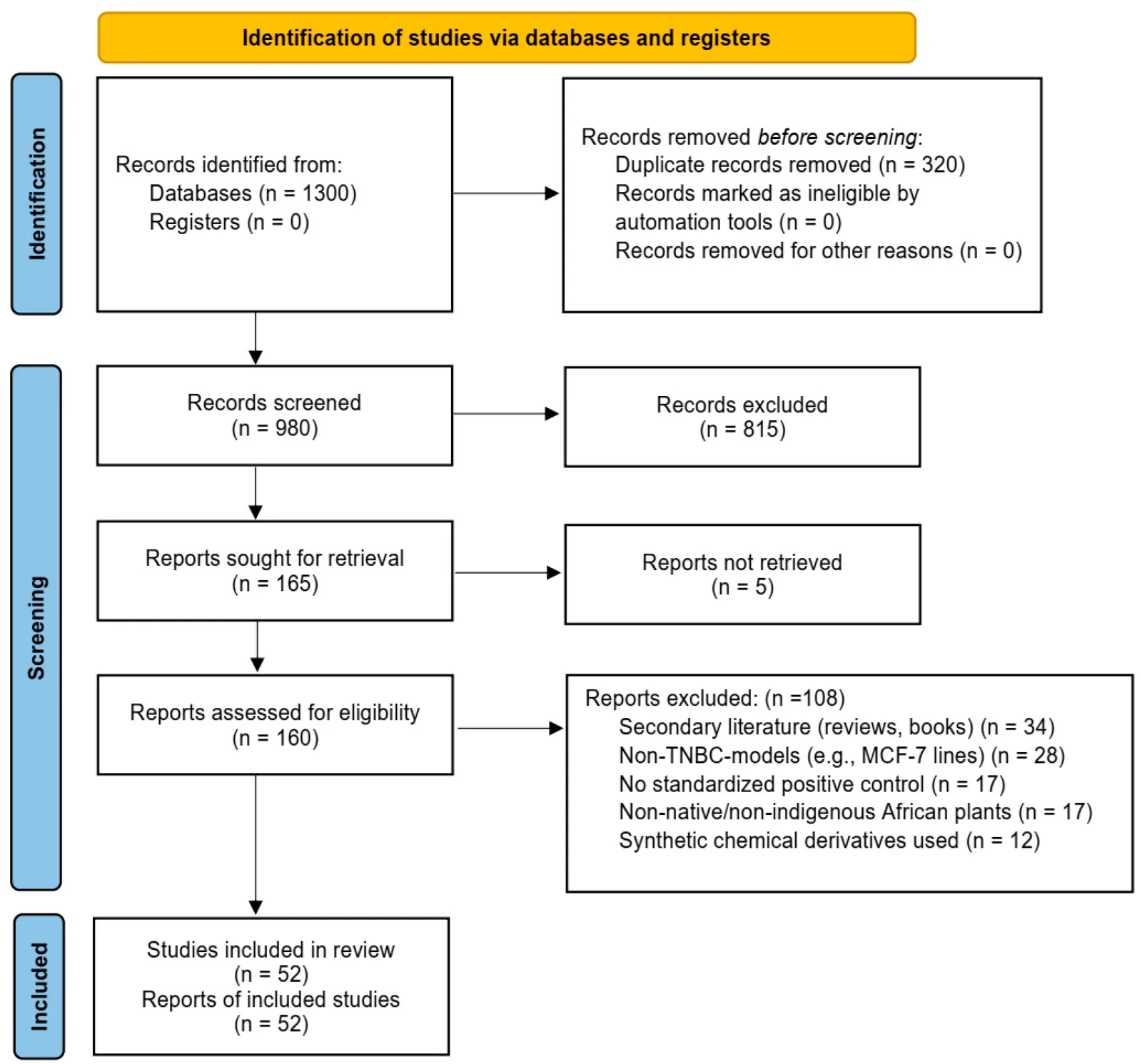
PRISMA 2020 flow diagram illustrating the systematic selection, screening, and inclusion process of literature evaluating African medicinal plants against triple-negative breast cancer (TNBC) models.

The Figure 4 further demonstrates the ability of medicinal plants to prevent metastasis, a major cause of cancer-related mortality. This is accomplished by reducing cancer cell migration and invasion, thereby impairing the ability of TNBC cells to disseminate from the primary tumor site to distant organs [109]. The suppression of metastatic potential is closely linked to the modulation of signalling pathways and enzymes involved in extracellular matrix degradation and cell motility.

Beyond these primary effects, the diagram highlights several downstream molecular and physiological processes influenced by medicinal plants. Notably, the inhibition of key oncogenic signalling pathways such as STAT3 and NF-κB is depicted. These pathways play crucial roles in promoting cancer cell survival, proliferation, inflammation, and immune evasion. Their suppression contributes significantly to the overall anticancer activity of medicinal plant-derived compounds. In parallel, anti-inflammatory effects are observed through the downregulation of pro-inflammatory cytokines, including interleukin-6 (IL-6) and tumor necrosis factor-alpha (TNF-α), which are known to support tumor progression and metastasis [112].

Another important mechanism illustrated in the Figure 3 is the inhibition of angiogenesis, primarily mediated through the suppression of vascular endothelial growth factor (VEGF). By preventing the formation of new blood vessels, medicinal plants effectively restrict the supply of oxygen and nutrients required for tumor growth and survival. Additionally, the enhancement of the immune response is depicted, with increased activity of immune effector cells such as T lymphocytes and natural killer (NK) cells. This immunomodulatory effect strengthens the body’s natural defense mechanisms against cancer cells.

Generally, these interconnected mechanisms culminate in the overall inhibition of TNBC tumor growth, as indicated at the base of the figure. The diagram emphasizes that medicinal plants exert their anticancer effects through a coordinated and multi-faceted approach, targeting various hallmarks of cancer simultaneously. This broad-spectrum activity underscores their potential as valuable sources of therapeutic agents for the management of TNBC and supports their continued exploration in cancer research and drug development [91,92,96].

## 3. Methodology

This was systematically done for the purpose of critical appraisal of literature concerning Africa natural flora and their metabolites anticancer properties against triple negative breast cancer. In other to meet the interpretative and conceptually integrative objectives, the systematic review on African medicinal plants targeting Triple-Negative Breast Cancer (TNBC) and, their ethnobotanical surveys, phytochemistry and anti-TNBC Studies was conducted in accordance with the Preferred Reporting Items for Systematic Reviews and Meta-Analyses (PRISMA) guidelines [145], and a PRISMA checklist was completed (Supplementary File S1).

### 3.1. Information Sources and Search Strategy

To ensure a comprehensive collection of data, a systematic search was conducted across multiple electronic databases, including PubMed, ScienceDirect, Scopus, web of science and African Journals OnLine (AJOL). The search strings were constructed using the following terms: For PubMed/Scopus/Web of Science/ScienceDirect: (“Medicinal plants” OR “Ethnobotany”) AND (“Africa” OR “African flora”) AND (“Triple-Negative Breast Cancer” OR “TNBC” OR “MDA-MB-231”) AND (“Phytochemistry” OR “Secondary metabolites”). For AJOL: (“Medicinal plants”) AND (“TNBC”) AND (“Anticancer activity”). Any discrepancies arising at any stage of the screening or eligibility phase were resolved through discussion. All searches were limited to peer-reviewed English-language publications published.

### 3.2. Study Identification and Selection Process

This systematic framework yielded an initial pool of 1300 publications spanning the years 2011 to 2026, distributed across the repositories. These initial publications comprised 979 original research articles, 220 patents, 58 reviews or book chapters, 32 clinical trials, and 11 conference papers. Overlapping and duplicate citations were filtered out using the Rayyan predictive platform (*Intelligent Systematic Review*, www.rayyan.ai) in February 2026, which automatically identified and removed 320 duplicate entries.

The remaining 980 unique records were subjected to an initial screening of titles and abstracts carried out independently by four reviewers (J.F.T.M., B.P.L., G.M.H., and A.O.A.). This screening step led to the exclusion of 815 irrelevant records. Following this initial screening, 165 reports were sought for retrieval; 160 full-text articles were successfully secured, while 5 reports could not be retrieved due to absolute source unavailability.

During the exhaustive full-text evaluation of these 160 secured papers, 108 articles were further excluded based on strict pre-defined criteria, leaving a final set of 52 primary articles for synthesis. Specifically, papers were excluded because they constituted secondary literature (review articles, editorials, conference abstracts, books, and book chapters), focused on general breast cancer without targeting the specific Triple-Negative subtype (e.g., evaluating effects exclusively on estrogen-receptor-positive MCF-7 cell models), lacked a standardized chemotherapy positive control, investigated plant species not native or indigenous to Africa or involved synthetic chemical derivatives not directly isolated from African flora. Any discrepancies arising at any screening stage were resolved through collective discussion and consensus with the senior team (J.F.T.M. and E.N.N.).

Finally, the protocol for this systematic review has been formally submitted to the International Prospective Register of Systematic Reviews (PROSPERO) and is currently undergoing the platform’s standard review process for the assignment of a registration ID. To ensure transparency and prevent retrospective bias, the investigation strictly adhered to the pre-defined methodological criteria and structural PRISMA steps outlined throughout this section. In Figure 4, the flow chart diagram of study enrollment is depicted.

### 3.3. Inclusion and Exclusion Criteria

The search was strictly focused on original, peer-reviewed, non-clinical laboratory research evaluating the anti-TNBC potential of extracts and pure secondary metabolites isolated from medicinal plants native to the African continent. This literature retrieval was limited to articles published in English within a strict multi-year window from 2011 to 2026. Studies utilized established in vitro TNBC cell models, such as MDA-MB-231, BT-20, HCC1937, or 4T1 lines, provided they explicitly compared the plant-derived fractions with a standard chemotherapy positive control. Crucially, to ensure high mechanistic rigor, candidate papers were required to conduct explicit evaluations of specific downstream oncogenic targets; hence, investigations were exclusively included if they reported cellular and transcriptomic indicators, including apoptosis induction (Caspases, Bcl-2/Bax pathways), cell cycle distribution checkpoints, gene expression profiles via quantitative PCR (RT-qPCR), and key protein signalling networks such as the PI3K/Akt/mTOR or MAPK cascades.

### 3.4. Study Selection and Data Extraction

The multi-tiered screening and selection pipeline strictly followed the PRISMA (Preferred Reporting Items for Systematic Reviews and Meta-Analyses) guidelines. Initially, titles and abstracts were screened for relevance. Potentially eligible studies underwent a full-text review by the authors. This narrowed the field with the potentially eligible studies underwent a rigorous full-text review leading to the 160 retrieved papers by the authors. Data were extracted into a standardized Excel spreadsheet including botanical information: Botanical name, family, local name, and geographical location which collectively yielded a total of 30 distinct genera distributed across 22 botanical families. Ethnobotanical Data: Plant part used (leaves, roots, bark, etc.) and mode of preparation. Phytochemistry: Main classes of compounds identified (alkaloids, terpenoids, polyphenols) and specific isolated molecules. Finally, detailed pharmacological activities were extracted, explicitly focusing on the type of extraction solvent used and the exact IC_50_ threshold values.

### 3.5. Quality Assessment and Risk of Bias Evaluation

To assess the methodological quality and risk of bias of the included in vitro studies, a tailored scoring matrix was developed by adapting standard rigorous guidelines to the specificities of natural product research. Each study was independently evaluated across six critical methodological domains: botanical authentication (verification of plant identity, family, and voucher specimen deposition), extract standardization (clear description of extraction solvents, yields, and chemical profiling or fingerprinting), cell line conditions and authentication (specification of origins, passage numbers, culture media, and STR profiling/mycoplasma status), experimental replication (performance of a minimum of three independent biological replicates with clear statistical analysis), and methodological controls (explicit inclusion of negative vehicle controls and standardized chemotherapy positive controls), and selectivity assessment (evaluation against non-cancerous cells to calculate a selectivity index)**.** Each domain was scored as 2 (fully reported/low risk of bias), 1 (partially reported/moderate risk of bias), or 0 (not reported/high risk of bias), yielding a maximum possible score of 12 points per study, where total scores of ≥9, 6–8, and <6 were categorized as low risk (high quality), moderate risk, and high risk (low quality), respectively (Table 4).

## 4. Conclusions and Recommendations

Breast cancer remains one of the leading health threats globally, with its trends in incidence and mortality rates continuing to persist and requiring further improvements in treatment approaches. Treatments such as chemotherapy, radioactive cancer therapy, and surgery are effective; however, they often have severe adverse effects that significantly impact patients’ quality of life. Triple-Negative Breast Cancer (TNBC) molecular profile limits the effectiveness of targeted therapies and contributes to its aggressive clinical behaviour. More so, treatment outcomes of TNBC are often compromised by the rapid emergence of drug resistance.

The catalog of botanical species documented across various African regions highlights an immense therapeutic promise for oncological management. Taxa such as *Nauclea pobeguinii*, *Phyllanthus muellerianus*, *Xylopia aethiopica*, and *Acacia erioloba* are among the plants with purported anticancer properties that remain under-researched, thus requiring more intensive scientific scrutiny to validate their traditional claims.

Furthermore, it is essential to broaden the scope of ethnobotanical field studies into under-represented geographical zones to discover additional candidate plants with antineoplastic potential. Species like *Vernonia amygdalina*, which is already recognized for its versatile antimalarial and antidiabetic applications, should be prioritized for mechanistic evaluation against Triple-Negative Breast Cancer (TNBC) cell lines in an upcoming specialized investigation.

A critical gap identified in this systematic review is the lack of toxicity screening against normal mammary epithelial cells (e.g., MCF-10A) across the included primary literature. Most studies focused strictly on evaluating cytotoxic efficacy against aggressive TNBC phenotypes (such as MDA-MB-231, BT-20, HCC1937, or 4T1), leaving the Selectivity Index (SI) of many extracts undetermined. Standardizing the inclusion of non-tumorigenic control cell lines is imperative for future African ethnopharmacological research to ensure therapeutic safety alongside anti-TNBC potency.

In essence, shifting from traditional usage to standardized clinical applications requires a rigorous focus on pharmacokinetic profiling and safety assessments. African health authorities should support the creation of regional drug development centers to transform this ancestral knowledge into accessible, evidence-based therapies for the continent.

## Figures and Tables

**Table 1:** Anti-TNBC cytopharmacological profiles of crude extracts and fractions from African medicinal plants.

Plant	Parts Used	Solvent	Cell Line	Assay	Time	IC_50_ Values	Selectivity Index	Positive Control	Mechanistic Evidence Level (TNBC Specificity)	Ref.
*Tulbaghia violacea*	Leaves	Water Methanol	MDA-MB-231	MTT/WST-1	48/72 h	314 to 400 µg/mL 780 to 820 µg/mL	Not reported	Doxorubicin	Level 1	[17,18]
*Catharanthus roseus*	Leaves	Methanol	MCF-7/T47D	SRB Assay	48 h	57.64 µg/mL	Not reported	Vinblastine	Level 2	[19,20]
*Myrothamnus flabellifolius*	Leaves, Stems	Methanol Ethyl Acetate	MDA-MB-231	XTT Assay	72 h	60 µg/mL ~50 µg/mL	Not reported	Paclitaxel	Level 1	[21,22]
*Vachellia erioloba*	Leaflets	Methanol	MCF-7/T47D	MTT Assay	48 h	Not reported	Not reported	5-Fluorouracil	Level 2	[23]
*Centella asiatica*	Leaves	Methanol	MDA-MB-231	MTT Assay	48 h	648 µg/mL	Not reported	Cisplatin	Level 1	[24]
*Crinum zeylanicum*	Bark	Dichloromethane	MDA-MB-231	Resazurin	48 h	44.75 µg/mL	Not reported	Doxorubicin	Level 1	[25]
*Curcuma longa*	Rhizomes	Ethanol/Methanol	MDA-MB-231	WST-1 Assay	48 h	13.7 µg/mL 2 µg/mL	Not reported	Doxorubicin	Level 1	[26]
*Nigella sativa*	Whole plant	Ethanolic	MDA-MB-231	MTT Assay	72 h	12.5 µg/mL	Not reported	Paclitaxel	Level 1	[27]
*Elephantopus scaber*	Whole plant	Ethanolic/Fractions	MDA-MB-231	SRB Assay	48 h	15 to 50 µg/mL	Not reported	Doxorubicin	Level 1	[28]
*Nauclea pobeguinii*	Bark	Methanol	MDA-MB-231	MTT Assay	72 h	30 µg/mL 11 µg/mL	Not reported	Doxorubicin	Level 1	[29,30]
*Xylopia aethiopica*	Seeds, fruits and Roots	Ethyl Acetate, Ethanol, or Diethyl Ether	MD-MB-231/HCC-70	MTT Assay	48 h	20.83 ± 1.05 µg/mL 19.96 µg/mL	Not reported	Paclitaxel	Level 1	[31,32]
*Annona muricata*	Leaves, Seeds	Methanolic/Ethyl acetate	MDA-MB-231	MTT Assay	48 h	200 µg/mL 10 µg/mL 0.128 ± 0.03 to 18.03 ± 0.20 µg/mL	Not reported	Doxorubicin	Level 1	[33,34,35,36,37]
*Punica granatum*	Peel, Seeds, Juice	MeOH/EtOH/Aq.	MDA-MB-231	MTT Assay	72 h	100 to 250 µg/mL	Not reported	Tamoxifen	Level 1	[38]
*Sutherlandia frutescens*	Leaves, Stems	EtOH/MeOH/Aq.	MCF-7/T47D	WST-1 Assay	48 h	2–10 mg/mL	Not reported	Doxorubicin	Level 2	[39]
*Vernonia amygdalina*	Leaves	Ethyl acetate Dichloromethane	MDA-MB-231/Hs578t	Crystal Violet	48/72 h	40.41 ± 0.49 µg/mL/4.57 ± 0.22 µg/mL 88 µg/mL	Not reported	Doxorubicin	Level 1	[40,41,42]
*Azadirachta indica*	Leaves, Flowers, Bark, Seeds	Methanolic/EtOH	MDA-MB-231	MTT Assay	48 h	62.5 µg/mL	Not reported	Cisplatin	Level 1	[43]
*Melia azedarach*	Stem bark	Methanol/Ethyl Acetate	MDA-MB-231	MTT Assay	48 h	62.2 to 172.9 µg/mL	Not reported	Doxorubicin	Level 1	[44]
*Maclura pomifera*	Leaves, Fruits	Methanol	MCF-7	SRB Assay	72 h	47.77 µg/mL	Not reported	Tamoxifen	Level 2	[45]
*Silybum marianum*	Seeds	Ethanol, Methanol, Ethyl acetate	MCF-7/T47D	MTT Assay	48 h	25 to 100 µg/mL	Not reported	Doxorubicin	Level 2	[46,47]
*Viscum album*	Leaves, Stems, and Berries	Water (Aqueous), Ethanol (Hydro-alcoholic)	MCF-7/T47D	Caspase Assay	48 h	20 µg/mL to 100 µg/mL	Not reported	Doxorubicin	Level 2	[48,49]
*Withania somnifera*	Roots and Leaves	Ethanol, Methanol	MDA-MB-231	XTT Assay	72 h	30 to 50 µg/mL	Not reported	Doxorubicin	Level 1	[50,51]
*Artemisia annua*	Aerial parts	DCM/Aq./Hydro-EtOH	MDA-MB-231	MTT Assay	48 h	2.0 to 40 µg/mL	Not reported	Paclitaxel	Level 1	[52,53,54]
*Bupleurum rotundifolium* L.	Leaves, Stems, Seeds	Aqueous/Hydro-EtOH	MCF-7/T47D	MTT Assay	48 h	20 to 320 µg/mL	Not reported	5-Fluorouracil	Level 2	[55]
*Peganum harmala*	Seeds	Aqueous	MDA-MB-231	MTT Assay	48 h	0.18 to 15.65 µg/mL	Not reported	Cisplatin	Level 1	[56,57]
*Moringa oleifera*	Leaves, pods, seeds, bark	MeOH/Water/EtOH	MDA-MB-231	MTT Assay	72 h	~100 to ~200 µg/mL 134.4 to 294.6 µg/mL	Not reported	Doxorubicin	Level 1	[58,59,60,61]
*Zanthoxylum zanthoxyloides*	Root, Stem, bark, fruits	Methanol	MDA-MB-231/BT-549	MTT Assay	48 h	55 µg/mL	Not reported	Doxorubicin	Level 1	[62,63,64]
*Phyllanthus muellerianus*	Leaves and Stem Bark	Methanol or Aqueous	MDA-MB-231	MTT Assay	48 h	~1.7 µg/mL	Not reported	Doxorubicin	Level 1	[65]
*Hypoxis hemerocallidea*	Corms (Rhizomes)	Methanol or Aqueous	MCF-7/T47D	MTT Assay	48 h	44.82 to 63.1 µg/mL 55.02 to 57.6 µg/mL	Not reported	Doxorubicin	Level 2	[66,67]
*Garcinia kola Heckel*	Seeds (Nuts) or Stem Bark	Methanol or Ethanol	MDA-MB-231	MTT Assay	48 h	~6.25 to 40 µg/mL	Not reported	Doxorubicin	Level 1	[68,69]
*Aloe ferox Mill*	Leaves	Methanol, Ethanol, or Ethyl Acetate	MCF-7/T47D	MTT Assay	48 h	~54.98 to 57.35 μg/mL	Not reported	Doxorubicin	Level 2	[70]

Cell Line Tested: MDA-MB-23, T47D, BT-20, SUM159, BT-549, 4T1, Hs578t.

**Table 2:** In vitro anti-TNBC pharmacokinetic and phenotypic indicators of isolated secondary metabolites from African flora.

Compound	Chemical Class	Source Plant	Cell Line	Assay	Time	IC_50_ in µM or nM	Molecular Target	Selectivity Index	Mechanistic Evidence Level [TNBC Specificity]	Ref.
Leurosine	Alkaloid	*Catharanthus roseus*	MDA-MB-231	SRB Assay	48 h	0.73 ± 0.06 µM	Tubulin polymerization	Not reported	Level 1	[20]
3,4,5-tri-O-galloylquinic acid Strictinin	Tannin Ellagitannin	*Myrothamnus flabellifolius*	MDA-MB-231/BT-549	AlamarBlue	72 h	30 µg/mL 30 µM	ROR1 receptor/PI3K	High Selective	Level 1	[21,22,71]
Rutin	Flavonoid	*Vachellia erioloba*	MCF-7/T47D	MTT Assay	48 h	137 ± 6.2 μM	c-Met Oncoprotein	Not reported	Level 2	[23]
Asiatic acid Asiaticoside	Triterpenoid Saponin	*Centella asiatica*	MDA-MB-231	MTT Assay	24 h	8, 12 µM ~48 µM	MAPK/ERK pathway	Not reported	Level 1	[24,72]
Crinine	Alkaloid	*Crinum zeylanicum*	MDA-MB-231	Resazurin	48 h	14.04 µM	Apoptotic disassembly	Not reported	Level 1	[25]
Curcumin	Curcuminoid	*Curcuma longa*	MDA-MB-231/4T1	WST-1 Assay	48 h	~15–30 µM	NF-κB/STAT3 Axis	Not reported	Level 1	[73]
Thymoquinone α-hederin	Quinone Saponin	*Nigella sativa*	MDA-MB-231	MTT Assay	72 h	25.37 µM 27.39 µM 10 µM	Bcl-2/Bax balance	Not reported	Level 1	[74,75]
Deoxyelephantopin Isodeoxyelephantopin	Sesquiterpene lactone Sesquiterpene lactone	*Elephantopus scaber*	MCF-7/T47D	SRB Assay	48 h	10 µM ≈9.5 μM	STAT3 signaling cascade	Not reported	Level 2	[76,77]
Resveratrol β-D-glucopyranoside Strictosamide	Stilbene Alkaloid	*Nauclea pobeguinii*	MDA-MB-231/BCRPM	MTT Assay	72 h	85.53 µM	Collateral sensitivity	Not reported	Level 1	[29,30]
Isotetrandrine	Alkaloid	*Xylopia aethiopica*	MDA-MB-231/HCC-70	ATP Assay	48 h	1.45–7.28 μM	Caspase activation	Not reported	Level 1	[78]
Annonacin	Acetogenin	*Annona muricata*	MDA-MB-231	MTT Assay	48 h	8.5 µM 15 µM	Mitochondrial Complex I	Not reported	Level 1	[79]
Punicic acid Punicalagin	Fatty acid Ellagitannin	*Punica granatum*	MDA-MB-231	WST-8 Assay	72 h	10–50 µM 40 µM to 50 µM	Autophagic pathways	Not reported	Level 1	[80,81]
L-Canavanine	Amino acid	*Sutherlandia frutescens*	MCF-7/T47D	WST-1 Assay	48 h	≈25 à 50 µg/mL	Mitochondrial disruption	Not reported	Level 2	[82]
Vernodaline Vernonioside B1	Sesquiterpene lactone Saponin	*Vernonia amygdalina*	MDA-MB-231 MCF-7	Crystal Violet	48 h	<15 µM 20–50 µM	PI3K/Akt signaling	Not reported	Level 1 and 2	[83]
Nimbolide	Limonoid	*Azadirachta indica*	MDA-MB-231	MTT Assay	48 h	2 µM to 12 µM	Caspase-dependent death	Not reported	Level 1	[43]
Meliazedin C	Limonoid	*Melia azedarach*	MDA-MB-231	MTT Assay	48 h	0.035 to 5.583 µM	Lysosomal acidification	Not reported	Level 1	[44,84]
Pomiferin Osajin	Isoflavone Isoflavone	*Maclura pomifera*	MDA-MB-231	SRB Assay	72 h	5.2 µM	TRPV1 Channel activation	Determined (MCF-10A)	Level 1	[45]
Silibinin	Flavonolignan	*Silybum marianum*	MDA-MB-231/Hs578T	MTT Assay	48 h	100 µM to 200 µM	EMT regulators/STAT3	Not reported	Level 1	[46,47,85]
Agglutinin (VCA Lectins)	Lectin/Biologic	*Viscum album*	MDA-MB-231	Caspase-Glo	48 h	~4.2 ng/mL (2D) ~12.8 ng/mL (3D)	Not reported	Not reported	Level 1	[48,49,86]
Withaferin A	Withanolide	*Withania somnifera*	MDA-MB-231/BT-549, SUM159	XTT Assay	72 h	~1.06 µM	EGFR/PI3K/Akt/mTOR axes	Not reported	Level 1	[50,51]
Chrysosplenol D Artemisinin Casticin	Flavonoid Sesquiterpene lactone Flavonoid	*Artemisia annua*	MDA-MB-231/BT-20	MTT Assay	48 h	22.1 µM/26.6 µM 25 µM (at I > 100) 20–40 µM	ERK1/2 pathway activation	Not reported	Level 1	[52,53,54]
Saikosaponin D	Saponin	*Bupleurum rotundifolium* L.	MCF-7/T47D	MTT Assay	48 h	10.2 µM	Mitochondrial apoptotic core	Not reported	Level 2	[55,87]
Harmine/Harmaline	Alkaloid	*Peganum harmala*	MDA-MB-231	MTT Assay	48 h	5.43 µg/mL	Topoisomerase inhibition	Not reported	Level 1	[56,57]
Quercetin E0771	Flavonoid	*Moringa oleifera*	MDA-MB-231	MTT Assay	72 h	20 µM to ~150 µM	VEGF Angiogenesis axis	Not reported	Level 1	[58,59,60,61]
Dihydrochelerythrine Sesamin	Alkaloid Lignan	*Zanthoxylum zanthoxyloides*	BT-549/MDA-MB-231	MTT Assay	48 h	21.2 µM 47.6 µM	Pro-apoptotic induction	Not reported	Level 1	[62,63,64]
Geraniin	Tannin	*Phyllanthus muellerianus*	MDA-MB-231	MTT Assay	48 h	~12.5 to 40 µM	Executioner caspase activation	Not reported	Level 1	[65]
Rooperol	Norlignan	*Hypoxis hemerocallidea*	MCF-7/T47D	MTT Assay	48 h	~15.0 to 30.0 μM	Mitochondrial stress	Not reported	Level 2	[66,67,88]
Garcinol Kolaviron	Benzophenone Biflavonoid	*Garcinia kola Heckel*	MDA-MB-231	MTT Assay	48 h	~1.56 to 25 μM	NF-κB/ROS modulation	Not reported	Level 1	[68,69]
Aloin Aloe-emodin	Anthraquinone	*Aloe ferox Mill*	MCF-7/T47D	MTT Assay	48 h	~1.15 mg/mL ~15 to 50 μM	NF-κB/Cell cycle arrest	Not reported	Level 2	[70,89]

Cell Line Tested: MDA-MB-23, T47D, BT-20, SUM159, BT-549, 4T1, Hs578t.

**Table 3:** Detailed study-by-study Risk of Bias (RoB) and methodological quality assessment.

Included Plant Species	B	S	C	R	M	Sl	Total Score	Risk of Bias
*Tulbaghia violacea*	?	+	?	+	?	−	7/12	Moderate Risk
*Catharanthus roseus*	+	+	+	?	+	?	10/12	Low Risk
*Myrothamnus flabellifolius*	?	?	?	+	?	−	6/12	Moderate Risk
*Vachellia erioloba*	?	+	?	+	?	?	8/12	Moderate Risk
*Centella asiatica*	+	+	?	+	+	?	10/12	Low Risk
*Crinum zeylanicum*	?	?	?	?	?	−	5/12	Moderate Risk
*Curcuma longa*	+	+	?	+	+	?	10/12	Low Risk
*Nigella sativa*	+	+	?	+	+	?	10/12	Low Risk
*Elephantopus scaber*	?	?	?	+	?	?	7/12	Moderate Risk
*Nauclea pobeguinii*	?	+	?	+	?	?	8/12	Moderate Risk
*Xylopia aethiopica*	?	+	?	+	?	?	8/12	Moderate Risk
*Annona muricata*	+	+	?	+	+	?	10/12	Low Risk
*Punica granatum*	+	+	?	+	+	?	10/12	Low Risk
*Prunus africana*	?	+	?	+	?	?	8/12	Moderate Risk
*Sutherlandia frutescens*	?	+	?	+	?	?	8/12	Moderate Risk
*Vernonia amygdalina*	+	+	?	+	+	?	10/12	Low Risk
*Azadirachta indica*	?	+	?	+	?	?	8/12	Moderate Risk
*Maclura pomifera*	?	?	?	?	?	?	6/12	Moderate Risk
*Silybum marianum*	?	?	?	+	?	?	7/12	Moderate Risk
*Viscum album*	?	+	?	+	?	?	8/12	Moderate Risk
*Withania somnifera*	+	+	?	+	+	?	10/12	Low Risk
*Artemisia annua*	+	+	?	+	+	?	10/12	Low Risk
*Bupleurum rotundifolium* L.	?	?	?	+	?	?	7/12	Moderate Risk
*Peganum harmala*	?	?	?	+	?	?	7/12	Moderate Risk
*Moringa oleifera*	+	+	?	+	+	?	10/12	Low Risk
*Zanthoxylum zanthoxyloides*	?	+	?	+	?	?	8/12	Moderate Risk

RoB Criteria Legend: B: Botanical Authentication & Voucher Specimen; S: Extract Extraction & Phytochemical Standardization; C: Cell Line Identification & Authentication Status; R: Experimental Replication & Statistical Rigor; M: Methodological Controls (Negative Vehicle & Standard Chemotherapy Positive Controls); Sl: Selectivity Index (Parallel evaluation against non-cancerous/normal cell lines). Risk Scoring Symbols: + Low Risk (Score 2); ? Moderate Risk (Score 1); − High Risk (Score 0).

**Table 4:** Methodological quality and risk of bias assessment criteria for the included in vitro studies.

Assessment Domain	Score 2 (Low Risk)	Score 1 (Moderate Risk)	Score 0 (High Risk)
Botanical Authentication	Full scientific name, family, author citation, and official voucher specimen number provided.	Plant name and part used mentioned, but missing voucher specimen data.	Vague or missing botanical identification.
Extract Standardization	Clear description of solvent, extraction method, yield, and chemical profiling (HPLC, NMR, etc.).	Extraction method mentioned, but lacking chemical profiling or characterization.	No details on extraction process or solvent composition.
Cell Line and Authentication	Specific cell lines named (e.g., MDA-MB-231), source/origin, and formal authentication (STR profiling/mycoplasma test) detailed.	Cell lines named with culture conditions, but missing formal authentication or origin data.	Vague description of cell models used.
Experimental Replication	At least 3 independent biological replicates performed with detailed statistical significance (*p*-values).	Replicates mentioned, but lack clear statistical analysis or error bars.	No mention of replication or statistical design.
Methodological Controls	Explicit inclusion of both negative vehicle controls (e.g., DMSO) and standardized positive chemotherapy controls.	Only negative controls or non-standard positive controls included.	Complete absence of appropriate experimental controls.
Selectivity Assessment	Cytotoxicity parallelly evaluated on non-cancerous/normal cell lines to calculate a Selectivity Index (SI).	Selectivity mentioned or implied, but no non-cancerous cell line included in the identical assay.	No evaluation of toxicity against non-cancerous cells.

## Data Availability

No new data were created or analyzed in this study. Data sharing is not applicable to this article.

## Supplementary Materials

The following supporting information can be downloaded at: https://www.mdpi.com/article/10.3390/ph19081134/s1, Supplementary File S1: PRISMA 2020 Checklist Table.

## Abbreviations

The following abbreviations are used in this manuscript:

DDMP	3,5-dihydroxy-6-methyl-4H-pyran-4-one
PARP	(Poly(ADP-ribose) polymerase)
ROS	Reactive oxygen species
TNBC	Triple Negative Breast Cancer

## References

[1] Wu R.R., Li X., Cao Y.H., Peng X., Liu G.-F., Liu Z.K., Yang Z., Liu Z.Y., Wu Y. (2023). China Medicinal Plants of the *Ampelopsis grossedentata*—Their Botanical Characteristics, Use, Phytochemistry, Active Pharmacological Components, and Toxicology. Molecules.

[2] Lehmann B.D., Bauer J.A., Chen X., Sanders M.E., Chakravarthy A.B., Shyr Y., Pietenpol J.A. (2011). Identification of human triple-negative breast cancer subtypes and preclinical models for selection of targeted therapies. J. Clin. Investig..

[3] Adrada B.E., Moseley T.W., Kapoor M.M., Scoggins M.E., Patel M.M., Perez F., Nia E.S., Khazai L., Arribas E., Rauch G.M. (2023). Triple-Negative Breast Cancer: Histopathologic Features, Genomics, and Treatment. RadioGraphics.

[4] Schmid P., Cortes J., Dent R., Pusztai L., McArthur H., Kümmel S., Bergh J., Denkert C., Park Y.H., Hui R. (2022). Event-free Survival with Pembrolizumab in Early Triple-Negative Breast Cancer. N. Engl. J. Med..

[5] Bardia A., Hurvitz S.A., Tolaney S.M., Loirat D., Punie K., Oliveira M., Brufsky A., Sardesai S.D., Kalinsky K., Zelnak A.B. (2021). Sacituzumab Govitecan in Metastatic Triple-Negative Breast Cancer. N. Engl. J. Med..

[6] Tutt A.N.J., Garber J.E., Kaufman B., Viale G., Fumagalli D., Rastogi P., Gelber R.D., de Azambuja E., Fielding A., Balmaña J. (2021). Adjuvant Olaparib for Patients with BRCA1- or BRCA2-Mutated Breast Cancer. N. Engl. J. Med..

[7] Park G.B., Kim D.J., Kim Y.S., Lee H.K., Kim C.W., Hur D.Y. (2015). Silencing of galectin-3 represses osteosarcoma cell migration and invasion through inhibition of FAK/Src/Lyn activation and β-catenin expression and increases susceptibility to chemotherapeutic agents. Int. J. Oncol..

[8] Yong L., Tang S., Yu H., Zhang H., Zhang Y., Wan Y., Cai F. (2022). The Role of Hypoxia-Inducible Factor-1 Alpha in Multidrug-Resistant Breast Cancer. Front. Oncol..

[9] Nwagu G.C., Bhattarai S., Swahn M., Ahmed S., Aneja R. (2021). Prevalence and mortality of triple-negative breast cancer in West Africa: Biologic and sociocultural factors. JCO Glob. Oncol..

[10] Innih S.O., Omage S.O., Lawal T.E., Omage K. (2022). Viscum Album Prevents Haematological Changes, Electrolyte Imbalance, Changes in Liver Function Enzymes and Histological Alterations in Some Selected Tissues in Cadmium Chloride-Intoxicated Rats. Saudi J. Biol. Sci..

[11] Motadi L.R., Choene M.S., Mthembu N.N. (2020). Anticancer properties of *Tulbaghia violacea* regulate the expression of p53-dependent mechanisms in cancer cell lines. Sci. Rep..

[12] Gouws C., Smit T., Willers C., Svitina H., Calitz C., Wrzesinski K. (2021). Anticancer potential of *Sutherlandia frutescens* and *Xysmalobium undulatum* in LS180 colorectal cancer mini-tumors. Molecules.

[13] Abdel-Sattar O.E., Allam R.M., Al-Abd A.M., El-Halawany A.M., El-Desoky A.M., Mohamed S.O., Sweilam S.H., Khalid M., Abdel-Sattar E., Meselhy M.R. (2023). Hypophyllanthin and phyllanthin from *Phyllanthus niruri* synergize doxorubicin anticancer properties against resistant breast cancer cells. ACS Omega.

[14] Caputi L., Franke J., Farrow S.C., Chung K., Payne R.M.E., Nguyen T.-D., Dang T.-T.T., Carqueijeiro I.S.T., Koudounas K., de Bernonville T.D. (2018). Missing enzymes in the biosynthesis of the anticancer drug vinblastine in Madagascar periwinkle. Science.

[15] Rafiu B.O., Omotayo A.O., Lawal I.O., Aremu A.O. (2025). Ethnobotanical uses of plants in Nigeria: An analysis of current research trends and patterns. J. Ethnobiol. Ethnomed..

[16] Salehi B., Quispe C., Imran M., Ul-Haq I., Živković J., Abu-Reidah I.M., Sen S., Taheri Y., Acharya K., Azadi H. (2021). Nigella Plants—Traditional Uses, Bioactive Phytoconstituents, Preclinical and Clinical Studies. Front. Pharmacol..

[17] Alaouna M., Molefi T., Khanyile R., Chauke-Malinga N., Chatziioannou A., Luvhengo T.E., Raletsena M., Penny C., Hull R., Dlamini Z. (2025). The Potential of the South African Plant *Tulbaghia violacea* Harv for the Treatment of Triple Negative Breast Cancer. Sci. Rep..

[18] Hull R., Dlamini Z., Penny C., Alaouna M. (2023). Abstract 3830: The Anticancer Activity of a Water-Soluble Extract from the Leaves of the Indigenous Southern African Plant *Tulbaghia violacea* against a Triple Negative Breast Cancer Cell Line. Cancer Res..

[19] Rajashekara S., Reena D., Mainavi M.V., Sandhya L.S., Baro U. (2022). Biological isolation and characterization of *Catharanthus roseus* (L.) G. Don methanolic leaves extracts and their assessment for antimicrobial, cytotoxic, and apoptotic activities. BMC Complement. Med. Ther..

[20] Wang C.H., Wang G.C., Wang Y., Zhang X.Q., Huang X.J., Zhang D.M., Chen M.F., Ye W.C. (2012). Cytotoxic dimeric indole alkaloids from *Catharanthus roseus*. Fitoterapia.

[21] Jaspal B., Norman F., Kayla A., Maria C.T., Bela P. (2018). A Novel Anti-Triple Negative Breast Cancer Compound Isolated from Medicinal Herb *Myrothamnus flabellifolius*. J. Med. Plants Res..

[22] Fultang N., Illendula A., Chen B., Wu C., Jonnalagadda S., Baird N., Klase Z., Peethambaran B. (2019). Strictinin, a Novel ROR1-Inhibitor, Represses Triple Negative Breast Cancer Survival and Migration via Modulation of PI3K/AKT/GSK3ß Activity. PLoS ONE.

[23] Batiha G.E.-S., Akhtar N., Alsayegh A.A., Abusudah W.F., Almohmadi N.H., Shaheen H.M., Singh T.G., de Waard M. (2022). Bioactive Compounds, Pharmacological Actions, and Pharmacokinetics of Genus Acacia. Molecules.

[24] Guo M., Ying Y., Chen Y., Miao X., Yu Z. (2024). Asiaticoside inhibits breast cancer progression and tumor angiogenesis via YAP1/VEGFA signal pathway. Heliyon.

[25] Wickramaratne N.S., Jeyaraj T., Senathilake K.S., Tennekoon K.H., Karunaratne D.N., Samarakoon S.R. (2024). Screening of Anti-Breast Cancer and Anti-Oxidant Potentials of Selected Medicinal Plants from Sri Lanka. Eur. J. Med. Plants.

[26] Sarkar E., Khan A., Ahmad R., Misra A., Raza S.T., Mahdi A.A. (2024). Synergistic anticancer efficacy of curcumin and doxorubicin combination treatment inducing S-phase cell cycle arrest in triple-negative breast cancer cells: An in vitro study. Cureus.

[27] Cheng L., Xia T., Wang Y., Zhou W., Liang X., Xue J., Shi L., Wang Y., Ding Q., Wang M. (2014). The anticancer effect and mechanism of α-hederin on breast cancer cells. Int. J. Oncol..

[28] Huang C.C., Lo C.P., Chiu C.Y., Shyur L.F. (2010). Deoxyelephantopin, a novel multifunctional agent, suppresses mammary tumour growth and lung metastasis and doubles survival time in mice. Br. J. Pharmacol..

[29] Kuete V., Sandjo L.P., Mbaveng A.T., Seukep J.A., Ngadjui B.T., Efferth T. (2015). Cytotoxicity of Selected Cameroonian Medicinal Plants and *Nauclea pobeguinii* towards Multi-Factorial Drug-Resistant Cancer Cells. BMC Complement. Altern. Med..

[30] Mesia G.K., Tona G.L., Penge O., Lusakibanza M., Nanga T.M., Cimanga R.K., Apers S., Van Miert S., Totte J., Pieters L. (2005). Antimalarial Activities and Toxicities of Three Plants Used as Traditional Remedies for Malaria in the Democratic Republic of Congo: *Croton mubango*, *Nauclea pobeguinii* and *Pyrenacantha staudtii*. Ann. Trop. Med. Parasitol..

[31] Alawode T.T., Odokwo E.O., Leoma M., Oderinlo O.O., Nyemba G.R., Davison C., Ngoepe M.P., de la Mare J.A., Tshiwawa T. (2026). Antiproliferative activity of *Xylopia aethiopica* extracts and molecular docking studies of their main phytochemicals. Silico Pharmacol..

[32] Kuete V., Sandjo L.P., Mbaveng A.T., Zeino M., Efferth T. (2015). Cytotoxicity of compounds from *Xylopia aethiopica* towards multifactorial drug-resistant cancer cells. Phytomedicine.

[33] Kim J.Y., Dao T.T.P., Song K., Park S.B., Jang H., Park M.K., Gan S.U., Kim Y.S. (2018). *Annona muricata* Leaf Extract Triggered Intrinsic Apoptotic Pathway to Attenuate Cancerous Features of Triple Negative Breast Cancer MDA-MB-231 Cells. *Evid. Based Complement*. Altern. Med..

[34] Pieme C.A., Kumar S.G., Dongmo M.S., Moukette B.M., Boyoum F.F., Ngogang J.Y., Saxena A.K. (2014). Antiproliferative activity and induction of apoptosis by *Annona muricata* (Annonaceae) extract on human cancer cells. BMC Complement. Altern. Med..

[35] Alshaeri H.K., Alasmari M.M., Natto Z.S., Pino-Figueroa A. (2020). Effects of *Annona muricata* Extract on Triple-Negative Breast Cancer Cells Mediated Through EGFR Signaling. Cancer Manag. Res..

[36] Yunani R., Sudjarwo S.A., Effendi M.H., Kurnijasanti R., Hidayah N. (2025). Annonacin Induces Apoptosis and Modulates Programmed Death-Ligand 1 and Interferon-Gamma Expression in Triple-Negative Breast Cancer: Integrated in Silico and in Vitro Analysis. Vet. World.

[37] Hernandez-Torres J., Guzman K.A. (2024). Abstract C026: Analyzing the effect of annonacin on diverse breast cancer cell lines as a potential therapeutic agent for triple negative breast cancer. Cancer Epidemiol. Biomark. Prev..

[38] Lim T.K. (2012). Punica granatum. Edible Medicinal and Non-Medicinal Plants: Volume 5, Fruits.

[39] Zonyane S., Fawole O.A., la Grange C., Stander M.A., Opara U.L., Makunga N.P. (2020). The implication of chemotypic variation on the anti-oxidant and anti-cancer activities of Sutherlandia frutescens (L.) R.Br. (Fabaceae) from different geographic locations. Antioxidants.

[40] Satria D., Hasibuan P.A.Z., Muhammad M., Waruwu S.B., Utomo R.Y., Ghoran S.H. (2024). Cytotoxic and Apoptotic Effect of *Vernonia amygdalina* Delile. Fractions against Hs578t Triple-Negative Breast Cancer Cell Lines. Phytomed. Plus.

[41] Hapsari N.P., Rahmawati D.R., Nugraheni N., Hanifa M., Satria D., Hasibuan P.A.Z., Meiyanto E. (2024). Africa leaf (*Vernonia amygdalina* Delille.) DCM extract synergistically supports growth suppression effect of doxorubicin on MCF7 and MCF7/HER2 with different effects on cell cycle progression and apoptosis evidence. J. Appl. Pharm. Sci..

[42] Wong F.C., Woo C.C., Hsu A., Tan B.K.H. (2013). The anti-cancer activities of *Vernonia amygdalina* extract in human breast cancer cell lines are mediated through caspase-dependent and p53-independent pathways. PLoS ONE.

[43] Jeelani I., Bhosale M., Qadir T., Sharma P.K., Nawaz A., Sharif A., Amin A., Sheikh A., Ahmad S., Kukreja V. (2023). Pharmaceutical Potential of Constituents from *Azadirachta indica* and their Specific Role as Anti-cancer Agents. Curr. Bioact. Compd..

[44] Zhang L., Ismail M.M., Rocchetti G., Fayek N.M., Lucini L., Saber F.R. (2022). The Untargeted Phytochemical Profile of Three Meliaceae Species Related to In Vitro Cytotoxicity and Anti-Virulence Activity against MRSA Isolates. Molecules.

[45] Yang R., Hanwell H., Zhang J., Tsao R., Meckling K.A. (2011). Antiproliferative activity of pomiferin in normal (MCF-10A) and transformed (MCF-7) breast epithelial cells. J. Agric. Food Chem..

[46] Mishra S.D., Mendonca P., Kaur S., Soliman K.F. (2025). Silibinin anticancer effects through the modulation of the tumor immune microenvironment in triple-negative breast cancer. Int. J. Mol. Sci..

[47] Binienda A., Ziolkowska S., Pluciennik E. (2020). The anticancer properties of Silibinin: Its molecular mechanism and therapeutic effect in breast Cancer. Anti Cancer Agents Med. Chem..

[48] Lyu S.Y., Meshesha S.M., Hong C.E. (2025). Synergistic Effects of Mistletoe Lectin and Cisplatin on Triple-Negative Breast Cancer Cells: Insights from 2D and 3D In Vitro Models. Int. J. Mol. Sci..

[49] Hong C.E., Lyu S.Y. (2026). Mistletoe Lectin Overcomes Macrophage-Mediated Chemo-Resistance in 3D Co-Culture Models of Triple-Negative Breast Cancer. Biocell.

[50] Srivastava A.N., Ahmad R., Khan M.A. (2015). Evaluation and Comparison of the In Vitro Cytotoxic Activity of *Withania somnifera* Methanolic and Ethanolic Extracts against MDA-MB-231 and Vero Cell Lines. Sci. Pharm..

[51] Mallipeddi H., Thyagarajan A., Sahu R.P. (2021). Implications of Withaferin-A for Triple-Negative Breast Cancer Chemoprevention. Biomed. Pharmacother..

[52] Lang S.J., Schmiech M., Hafner S., Paetz C., Werner K., El Gaafary M., Schmidt C.Q., Syrovets T., Simmet T. (2020). Chrysosplenol d, a Flavonol from *Artemisia annua*, Induces ERK1/2-Mediated Apoptosis in Triple Negative Human Breast Cancer Cells. Int. J. Mol. Sci..

[53] Agustina R., Ari P.M.S., Sadikin N.A.N. (2025). Anticancer Effectiveness of *Artemisia annua* Ethanol Extract against MDAMB-231 Cancer Cells. Int. J. Cell Biomed. Sci..

[54] Septembre-Malaterre A., Rakoto M.L., Marodon C., Bedoui Y., Nakab J., Simon E., Hoarau L., Savriama S., Strasberg D., Guiraud P. (2020). *Artemisia annua*, a Traditional Plant Brought to Light. Int. J. Mol. Sci..

[55] Liu W., Cheng X., Kang R., Wang Y., Guo X., Jing W., Wei F., Ma S. (2021). Systematic Characterization and Identification of Saikosaponins in Extracts From *Bupleurum marginatum var. stenophyllum* Using UPLC-PDA-Q/TOF-MS. Front. Chem..

[56] Hashemi Sheikh Shabani S., Seyed Hasan Tehrani S., Rabiei Z., Tahmasebi Enferadi S., Vannozzi G.P. (2015). *Peganum harmala* L.’s anti-growth effect on a breast cancer cell line. Biotechnol. Rep..

[57] Sedky N.K., Arafa K.K., Abdelhady M.M.M., Issa M.Y., Abdel-Kader N.M., Mahdy N.K., Mokhtar F.A., Alfaifi M.Y., Fahmy S.A. (2023). Nedaplatin/*Peganum harmala* Alkaloids Co-Loaded Electrospun, Implantable Nanofibers: A Chemopreventive Nano-Delivery System for Treating and Preventing Breast Cancer Recurrence after Tumorectomy. Pharmaceutics.

[58] Badhe P. (2025). *Moringa oleifera* in Breast Cancer: Targeting ROS, P53, and VEGF Pathways through Phytochemical Synergy. Biopress J. Comput. Life Sci. (BJCLS).

[59] Williams A. (2022). *Moringa oleferia* Extracts Inhibit Proliferation in Triple-Negative Breast Cancer Cell Lines. Master’s Thesis.

[60] Vijay P., Tamilselvi M., Mohankumar R. (2023). Isolation, identification and HPLC analysis of a phytochemical from *Moringa oleifera* leaves. Mater. Today Proc..

[61] Zunica E.R.M., Yang S., Coulter A., White C., Kirwan J.P., Gilmore L.A. (2021). *Moringa oleifera* Seed Extract Concomitantly Supplemented with Chemotherapy Worsens Tumor Progression in Mice with Triple Negative Breast Cancer and Obesity. Nutrients.

[62] Andima M., Coghi P., Yang L.J., Wong V.K.W., Ngule C.M., Heydenreich M., Ndakala A.J., Yenesew A., Derese S. (2019). Antiproliferative Activity of Secondary Metabolites from *Zanthoxylum zanthoxyloides* Lam: In Vitro and in Silico Studies. Pharmacogn. Commun..

[63] Guetchueng S.T., Nahar L., Ritchie K.J., Ismail F.M., Evans A.R., Sarker S.D. (2018). Zanthoamides GI: Three new alkamides from *Zanthoxylum zanthoxyloides*. Phytochem. Lett..

[64] Misra L.N., Wouatsa N.A.V., Kumar S., Venkatesh Kumar R., Tchoumbougnang F. (2013). Antibacterial, Cytotoxic Activities and Chemical Composition of Fruits of Two Cameroonian *Zanthoxylum* Species. J. Ethnopharmacol..

[65] Yaya C.D. (2023). Evaluation des propriétés anticancéreuses in vitro de *Phyllanthus muellerianus* (Phyllanthaceae), une plante utilisée dans le traitement du cancer du sein dans la région de l’Adamaoua (Cameroun). J. Afr. Technol. Pharm. Biopharm..

[66] Badeggi U.M., Omoruyi S.I., Ismail E., Africa C., Botha S., Hussein A.A. (2022). Characterization and Toxicity of Hypoxoside Capped Silver Nanoparticles. Plants.

[67] Kasumbwe K., Mohanlall V. (2025). In vitro cytotoxicity and phytochemical analysis of the crude extract from *Hypoxis hemerocallidea* and its apoptosis-inducing effect on breast and skin cancer. Braz. J. Biol..

[68] Origbemisoye B.A., Malomo S.A., Ifesan B.O. (2025). Impact of Processing and Extraction Methods on the Phytochemical Composition of *Garcinia kola* Stem Bark and Stone Breaker Leaves. Asian Food Sci. J..

[69] Alabi M.A., Muthusamy A., Kabekkodu S.P., Adebawo O.O., Satyamoorthy K. (2020). Anticancer properties of recipes derived from Nigeria and African medicinal plants on breast cancer cells in vitro. Sci. Afr..

[70] Sanders B., Ray A.M., Goldberg S., Clark T., McDaniel H.R., Atlas S.E., Farooqi A., Konefal J., Lages L.C., Lopez J. (2017). Anti-cancer effects of aloe-emodin: A systematic review. J. Clin. Transl. Res..

[71] Fultang N., Brar J., Mercier I., Klase Z., Peethambaran B. (2018). *Myrothamnus flabellifolius* Selectively Targets Triple Negative Breast Cancer in Vitro, Restoring Tamoxifen Sensitivity through Modulation of MiRNAs Associated with Estrogen Receptors. Int. J. Appl. Res. Nat. Prod..

[72] Hsu Y.L., Kuo P.L., Lin L.T., Lin C.C. (2005). Asiatic acid, a triterpene, induces apoptosis and cell cycle arrest through activation of extracellular signal-regulated kinase and p38 mitogen-activated protein kinase pathways in human breast cancer cells. J. Pharmacol. Exp. Ther..

[73] Hermansyah D., Paramita D.A., Paramita D.A., Amalina N.D. (2023). Combination *Curcuma longa* and *Phyllanthus niruri* Extract Potentiate Antiproliferative in Triple Negative Breast Cancer MDAMB-231 Cells. Asian Pac. J. Cancer Prev..

[74] Ma J., Peng C. (2024). Nigella sativa plant extract inhibits the proliferation of MDA-MB-231 breast cancer cells via apoptosis and cell cycle arrest. Bangladesh J. Pharmacol..

[75] Adinew G.M., Messeha S.S., Taka E., Badisa R.B., Antonie L.M., Soliman K.F.A. (2022). Thymoquinone Alterations of the Apoptotic Gene Expressions and Cell Cycle Arrest in Genetically Distinct Triple-Negative Breast Cancer Cells. Nutrients.

[76] Beeran A.A., Maliyakkal N., Rao C.M., Udupa N. (2015). The enriched fraction of *Elephantopus scaber* Triggers apoptosis and inhibits multi-drug resistance transporters in human epithelial cancer cells. Pharmacogn. Mag..

[77] Kabeer F.A., Sreedevi G.B., Nair M.S., Rajalekshmi D.S., Gopalakrishnan L.P., Prathapan R. (2014). Isodeoxyelephantopin from *Elephantopus scaber* (Didancao) induces cell cycle arrest and caspase-3-mediated apoptosis in breast carcinoma T47D cells and lung carcinoma A549 cells. Chin. Med..

[78] Soni S., Modi S.R., Andey T. (2024). Investigation of the antiproliferative effect of the *Xylopia aethiopica* extract in triple-negative breast cancer cells. Cancer Res..

[79] Kariyil B.J., Ayyappan U.P.T., Gopalakrishnan A., George A.J. (2021). Chloroform Fraction of Methanolic Extract of Seeds of *Annona muricata* Induce S Phase Arrest and ROS Dependent Caspase Activated Mitochondria-Mediated Apoptosis in Triple-Negative Breast Cancer. Anticancer Agents Med. Chem..

[80] Bagheri M., Fazli M., Saeednia S., Kor A., Ahmadiankia N. (2018). Pomegranate peel extract inhibits expression of β-catenin, epithelial mesenchymal transition, and metastasis in triple negative breast cancer cells. Cell. Mol. Biol..

[81] Bhutta Z.A., Go R.E., Choi K.C. (2024). Effect of Punicalagin on the Autophagic Cell Death in Triple-Negative Breast Cancer Cells. Toxicol. Res..

[82] Vorster C., Stander A., Joubert A. (2012). Differential signaling involved in Sutherlandia frutescens-induced cell death in MCF-7 and MCF-12A cells. J. Ethnopharmacol..

[83] Farombi E.O., Owoeye O. (2011). Antioxidative and chemopreventive properties of *Vernonia amygdalina* and *Garcinia biflavonoid*. Int. J. Environ. Res. Public Health.

[84] Nugraha A.S., Firli L.N., Hendra R., Keller P.A., Purwoko R.Y., Idrus H.H., Ritawidya R., Febrian M.B., Mahendra I., Kurniawan A. (2023). Anticancer activity of Indonesian *Melia azedarach* L.: Phyto-chemistry, in vitro and in silico studies. J. Biol. Act. Prod. Nat..

[85] Wang X., Zhang Z., Wu S.C. (2020). Health benefits of Silybum marianum: Phytochemistry, pharmacology, and applications. J. Agric. Food Chem..

[86] Hong C.E., Lyu S.Y. (2025). VCA Augments Doxorubicin Efficacy in Triple-Negative Breast Cancer: Evidence for Multi-Pathway Synergism. Biocell.

[87] Shahabadi N., Shokraei S., Shalmashi K., Soltani L. (2025). Green synthesis of *Bupleurum rotundifolium*–Silver nanoparticles: Characterization, biological activities, and preliminary environmental risk assessment. Ind. Crops Prod..

[88] Matyanga C.M.J., Morse G.D., Gundidza M., Nhachi C.F.B. (2020). African potato (*Hypoxis hemerocallidea*): A systematic review of its chemistry, pharmacology and ethno medicinal properties. BMC Complement. Med. Ther..

[89] Dastouri M., Sanli B. (2026). Aloin Induces Selective Cytotoxicity and Apoptotic Pathway Activation in Breast and Prostate Cancer Cells via Intrinsic and Extrinsic Mechanisms. Int. J. Mol. Sci..

[90] Lulekal E., Asfaw Z., Kelbessa E., Van Damme P. (2013). Ethnomedicinal Study of Plants Used for Human Ailments in Ankober District, North Shewa Zone, Amhara Region, Ethiopia. J. Ethnobiol. Ethnomed..

[91] Ochwang’I D.O., Kimwele C.N., Oduma J.A., Gathumbi P.K., Mbaria J.M., Kiama S.G. (2014). Medicinal Plants Used in Treatment and Management of Cancer in Kakamega County, Kenya. J. Ethnopharmacol..

[92] Misonge J.M., Kamindu G.N., Wangui S.W., Muita G.M., Onyancha J.M. (2019). An Ethnobotanical Survey of Plants Used for the Treatment and Management of Cancer in Embu County, Kenya. J. Med. Plants Stud..

[93] Kharchoufa L., Bouhrim M., Bencheikh N., Addi M., Hano C., Mechchate H., Elachouri M. (2021). Potential toxicity of medicinal plants inventoried in northeastern Morocco: An ethnobotanical approach. Plants.

[94] Semenya S.S., Potgieter M.J. (2014). Medicinal plants cultivated in Bapedi traditional healers homegardens, Limpopo Province, South Africa. Afr. J. Tradit. Complement. Altern. Med..

[95] Popoola J.O., Obembe O.O. (2013). Local knowledge, use pattern and geographical distribution of *Moringa oleifera* Lam. (Moringaceae) in Nigeria. J. Ethnopharmacol..

[96] Raimi I.O., Kopaopa B.G., Mugivhisa L.L., Lewu F.B., Amoo S.O., Olowoyo J.O. (2021). An ethnobotanical survey of medicinal plants used by traditional healers for the treatment of cancer in Hammanskraal and Winterveld, Tshwane Metropolitan Municipality, South Africa. Afr. Health Sci..

[97] Geisler S., Doan R.A., Strickland A., Huang X., Milbrandt J., DiAntonio A. (2016). Prevention of vincristine-induced peripheral neuropathy by genetic deletion of SARM1 in mice. Brain.

[98] Alam M.M., Naeem M., Khan M.M.A., Uddin M. (2017). Vincristine and Vinblastine Anticancer Catharanthus Alkaloids: Pharmacological Applications and Strategies for Yield Improvement. Catharanthus roseus.

[99] Taher Z.M., Agouillal F.L., Marof A.Q., Dailin D.J., Nurjayadi M., El Enshasy H.A. (2019). Anticancer Molecules from *Catharanthus roseus*. Indones. J. Pharm..

[100] Kuzminska J., Szyk P., Mlynarczyk D.T., Bakun P., Muszalska-Kolos I., Dettlaff K., Sobczak A., Goslinski T., Jelinska A. (2024). Curcumin derivatives in medicinal chemistry: Potential applications in cancer treatment. Molecules.

[101] Vel Szic K.S., de Beeck K.O., Ratman D., Wouters A., Beck I., Declerck K., Heyninck K., Fransen E., Bracke M., de Bosscher K. (2014). Pharmacological Levels of Withaferin A (*Withania somnifera*) Trigger Clinically Relevant Anticancer Effects Specific to Triple Negative Breast Cancer Cells. PLoS ONE.

[102] Lee I.C., Choi B. (2016). Withaferin-A—A Natural Anticancer Agent with Pleitropic Mechanisms of Action. Int. J. Mol. Sci..

[103] Bestari R., Ichwan M.F., Mustofa M., Satria D. (2018). Anticancer Activity of *Vernonia amygdalina* Del. Extract on WiDr Colon Cancer Cell Line. Proceedings of the 2nd Public Health International Conference (PHICo 2017).

[104] Chatri M., Hasibuan P.A., Meiyanto E., Putra D.P., Septisetyani E.P., Satria D., Waruwu S.B. (2024). Effect of African Leaves (*Vernonia amygdalina* Delile) on the Development of T47D Breast Cancer Cells. Trop. J. Nat. Prod. Res..

[105] Faisal M., Sukpondma Y., Sangket J., Taraporn S., Dokduang S., Graidist P. (2026). Vernodalin and *Gymnanthemum extensum* Crude Extracts Exhibit In Vitro Anticancer Activity with Differential Regulation of Cancer-Associated Signaling Proteins in Breast and Ovarian Cancer Cells. Biomedicines.

[106] Jain S., Panuganti V., Jha S., Roy I. (2020). Harmine Acts as an Indirect Inhibitor of Intracellular Protein Aggregation. ACS Omega.

[107] Berrougui H., Martincordero C., Khalil A., Hmamouchi M., Ettaib A., Marhuenda E., Herrera M. (2006). Vasorelaxant Effects of Harmine and Harmaline Extracted from *Peganum Harmala* L. Seeds in Isolated Rat Aorta. Pharmacol. Res..

[108] Alsaif G., Tasleem M., Rezgui R., Alshaghdali K., Saeed A., Saeed M. (2024). Network Pharmacology and Molecular Docking Analysis of *Catharanthus roseus* Compounds: Implications for Non-Small Cell Lung Cancer Treatment. J. King Saud Univ. Sci..

[109] Al-Amin M., Eltayeb N.M., Rahiman S.S.F., Khairuddean M., Salhimi S.M. (2022). UPLC-ESI-QTOF-MS/MS and 1H-NMR Identification of Alkaloids in Potent Fraction of *Catharanthus roseus* Leaves Inhibits Migration and Invasion of MDA-MB-231 Cells. Biologia.

[110] Deeh P.B.D., Arumugam M., Alagarsamy K., Karanam G., Natesh N.S., Watcho P., Vishwakarma V. (2022). *Phyllanthus muellerianus* and *Ficus exasperata* Exhibit Anti-Proliferative and pro-Apoptotic Activities in Human Prostate Cancer PC-3 Cells by Modulating Calcium Influx and Activating Caspases. Biologia.

[111] Zhang Y., Zhao Q., Sun X., Chen M., Wei Y., Zhang W., Ding X., Chen D., Hua H., Hao X. (2026). Meliazedins AH, limonoids with cytotoxicity and inhibitory activity on lysosomal acidification from *Melia azedarach* L. Fitoterapia.

[112] Termer M., Carola C., Salazar A., Keck C.M., Hemberger J., von Hagen J. (2021). Activity-Guided Characterization of COX-2 Inhibitory Compounds in *Waltheria indica* L. *Extracts*. Molecules.

[113] Listyo A.B., Kusrini D., Fachriyah E. (2018). Isolation of Phenolic Acid Compounds and Antioxidant Tests from Mindi Leaves (*Melia azedarach* L.). J. Kim. Sains Dan Apl..

[114] Ervina M., Poerwono H., Widyowati R., Otsuka H., Matsunami K., Sukardiman S. (2021). Pregnane Steroids from the Leaves of *Melia azedarach* and Apoptotic Activity against T47D Cells. Asian Pac. J. Cancer Prev..

[115] Ervina M., Poerwono H., Widyowati R., Matsunami K., Sukardiman S. (2020). Bio-Selective Hormonal Breast Cancer Cytotoxic and Antioxidant Potencies of *Melia azedarach* L. Wild Type Leaves. Biotechnol. Rep..

[116] Sumarawati T., Israhnanto Fatmawati D. (2017). Anticancer Mechanism of *Melia azedarach*, Doxorubicin and Cyclosphamide Combination against Breast Cancer in Mice. Bangladesh J. Med. Sci..

[117] Zhou L., Ou S., Liang T., Li M., Xiao P., Cheng J., Zhou J., Yuan L. (2023). MAEL facilitates metabolic reprogramming and breast cancer progression by promoting the degradation of citrate synthase and fumarate hydratase via chaperone-mediated au-tophagy. FEBS J..

[118] Rumpa M.M., Maier C. (2024). TRPV1-Dependent Antiproliferative Activity of Dioecious *Maclura pomifera* Extracts in Estrogen Receptor-Positive Breast Cancer Cell Lines Involves Multiple Apoptotic Pathways. Int. J. Mol. Sci..

[119] Saeed S., Khan M.I.A., Ayesha S., Zahid Z., Younis M., Kashan M.W., Khan G.Z., Tahir M., Khan S.A., Mansab T. (2023). Screening of Phytochemicals and Anticancer Potential of *Maclura pomifera* Leaves Extract. Pak. J. Med. Health Sci..

[120] Ribaudo G., Anyanwu M., Giannangeli M., Oselladore E., Ongaro A., Memo M., Gianoncelli A. (2024). G-Quadruplex DNA as a Macromolecular Target for Semi-Synthetic Isoflavones Bearing B-Ring Tosylation. Macromol.

[121] Dhillon J., Miller V., Carter J., Badiab A., Tang C.N., Huynh A., Peethambaran B. (2014). Apoptosis-Inducing Potential of *Myrothamnus flabellifolius*, an Edible Medicinal Plant, on Human Myeloid Leukemia HL-60 Cells. Int. J. Appl. Res. Nat. Prod..

[122] Ravhudzulo I., Mthana M.S., Ogwuegbu M.C., Ramachela K., Mthiyane D.M., Onwudiwe D.C. (2025). Phytogenic synthesis of zinc oxide nanoparticles using extract of *Vachellia erioloba* seed and their anticancer and antioxidant activity. Discov. Appl. Sci..

[123] Kuete V. (2024). Fighting colorectal cancer and its drug resistance with the resources of the flora of Africa. Advances in Botanical Research.

[124] Toona L.M., Mthombeni J., Mfengwana P.M.H. (2025). Cytotoxic activities of Hypoxis hemerocallidea, Helichrysum caespititium and Dicoma anomala extracts on DU145 prostate cancer cell line. J. Med. Plants Econ. Dev..

[125] Rahmawati D.R., Nugraheni N., Hapsari N.P., Hanifa M., Kastian R.F., Prasetyaningrum P.W., Satria D., Hasibuan P.A.Z., Septisetyani E.P., Meiyanto E. (2025). Dichloromethane Fraction of *Vernonia amygdalina* Delile Enhances the Cytotoxicity of Doxorubicin Against Triple-Negative Breast Cancer (TNBC) Cells Through Apoptosis Induction and Cell Cycle Modulation. Indones. J. Pharm..

[126] Howard C.B., Mcdowell R., Feleke K., Deer E., Stamps S., Thames E., Singh V., Pervin S. (2016). Chemotherapeutic Vulnerability of Triple-Negative Breast Cancer Cell-Derived Tumors to Pretreatment with *Vernonia amygdalina* Aqueous Extracts. Anticancer Res..

[127] Popoola T.D., Awodele O., Babawale F., Oguns O., Onabanjo O., Ibanga I., Godwin H., Oyeniyi T., Fatokun A.A., Akinloye O. (2020). Antioxidative, Antimitotic, and DNA-Damaging Activities of *Garcinia kola* Stem Bark, *Uvaria chamae* Root, and *Olax subscorpioidea* Root Used in the Ethnotherapy of Cancers. J. Basic Clin. Physiol. Pharmacol..

[128] Guetchueng S.T., Nahar L., Ritchie K.J., Sarker S.D. (2022). Evaluation of the Chemopreventive Effect of Selected Medicinal Plants Extracts via Induction of the Nrf2 in a Modified Model of Breast Cancer Cells: Identification of Bioactive Lead Compounds. Eur. J. Cancer Prev..

[129] Huang P.H., Huang C.Y., Chen M.C., Lee Y.T., Yue C.H., Wang H.Y., Lin H. (2013). Emodin and Aloe-Emodin Suppress Breast Cancer Cell Proliferation through ER α Inhibition. *Evid. Based Complement*. Altern. Med..

[130] Paramita D.A., Hermansyah D., Amalina N.D. (2022). Regulation of P53 and Survivin by *Curcuma longa* Extract to Caspase-3 Dependent Apoptosis in Triple Negative Breast Cancer Cells. Med. Glas..

[131] Zhou J., Guo Z., Peng X., Wu B., Meng Q., Lu X., Guo T. (2025). Chrysotoxine regulates ferroptosis and the PI3K/AKT/mTOR pathway to prevent cervical cancer. J. Ethnopharmacol..

[132] Ünal T.D., Hamurcu Z., Delibaşı N., Çınar V., Güler A., Gökçe S., Nurdinov N., Ozpolat B. (2020). Thymoquinone Inhibits Proliferation and Migration of MDA-MB-231 Triple Negative Breast Cancer Cells by Suppressing Autophagy, Beclin-1 and LC3. Anticancer Agents Med. Chem..

[133] Lintangsari G., Aliyah A.N., Maran G.G., Hermawan A., Meiyanto E. (2025). Black Seed Oil Inhibits the Migration of Triple-negative Breast Cancer Cells and Regulates MMP-9 Expression. Indones. J. Biotechnol..

[134] Kabil N., Bayraktar R., Kahraman N., Mokhlis H.A., Calin G.A., Lopez-Berestein G., Ozpolat B. (2018). Thymoquinone Inhibits Cell Proliferation, Migration, and Invasion by Regulating the Elongation Factor 2 Kinase (EEF-2K) Signaling Axis in Triple-Negative Breast Cancer. Breast Cancer Res. Treat..

[135] Lin D., Wang H., Wu S., Chen H. (2025). Isodeoxyelephantopin Promotes Cell Death in Triple-Negative Breast Cancer by Blocking STAT3 Phosphorylation and Enhancing the Effectiveness of Paclitaxel. Food Sci. Nutr..

[136] Berkov S., Romani S., Herrera M., Viladomat F., Codina C., Momekov G., Ionkova I., Bastida J. (2011). Antiproliferative alkaloids from Crinum zeylanicum. Phytother. Res..

[137] Dariushnejad H., Roshanravan N., Wasman H.M., Cheraghi M., Pirzeh L., Ghorbanzadeh V. (2024). Silibinin, Synergistically Enhances Vinblastine-Mediated Apoptosis in Triple Negative Breast Cancer Cell Line: Involvement of Bcl2/Bax and Caspase-3 Pathway. Int. J. Hematol. Oncol. Stem Cell Res..

[138] Shuaib M., Prajapati K.S., Gupta S., Kumar S. (2023). Natural Steroidal Lactone Induces G1/S Phase Cell Cycle Arrest and Intrinsic Apoptotic Pathway by Up-Regulating Tumor Suppressive MiRNA in Triple-Negative Breast Cancer Cells. Metabolites.

[139] Prasad K., Saggam A., Guruprasad K.P., Tillu G., Patwardhan B., Satyamoorthy K. (2024). Molecular Mechanisms of *Asparagus racemosus* Willd. and *Withania somnifera* (L.) Dunal as Chemotherapeutic Adjuvants for Breast Cancer Treatment. J. Ethnopharmacol..

[140] Lang S.J., Schmiech M., Hafner S., Paetz C., Steinborn C., Huber R., El Gaafary M., Werner K., Schmidt C.Q., Syrovets T. (2019). Antitumor Activity of an *Artemisia annua* Herbal Preparation and Identification of Active Ingredients. Phytomedicine.

[141] Ding Y., He J., Huang J., Yu T., Shi X., Zhang T., Yan G., Chen S., Peng C. (2019). Harmine induces anticancer activity in breast cancer cells via targeting TAZ. Int. J. Oncol..

[142] Alhawiti N.M., Myles E.L., Alhewaitey A.M., Almutairi Y.M., Alhawiti F.M. (2024). Cytotoxicity, Toxicity and Anticancer Activity of Manuka Honey, Saudi’s Honey and *Peganum harmala* Plant against Cancer Cells. J. Biosci. Med..

[143] Güzel M.A., Kolaç T., Menevşe İ.N., Dündar M., Zengin R., Güzel A., Uğur Y. (2026). Integrated chemical and biological characterization of Hypericum perforatum extract using LC-MS/MS and in vitro functional assays. Sci. Rep..

[144] Uğur Y., Menevşe İ.N., Dündar M., Karci H., Zengin R., Güzel A. (2025). Comparative chemical and biological evaluation of Urtica dioica extracts obtained by methanol and hexane: Antioxidant, cytotoxic, apoptotic, and antimicrobial potentials. BMC Complement. Med. Ther..

[145] Page M.J., McKenzie J.E., Bossuyt P.M., Boutron I., Hoffmann T.C., Mulrow C.D., Shamseer L., Tetzlaff J.M., Akl E.A., Brennan S.E. (2021). The PRISMA 2020 Statement: An Updated Guideline for Reporting Systematic Reviews. BMJ.

