# Supplementary material for: African Medicinal Plants Targeting Triple-Negative Breast Cancer (TNBC): A Systematic Review of Ethnobotanical Surveys, Phytochemistry and Anti-TNBC Studies
Source: Pharmaceuticals (Basel). 2026 Jul 23;19(8):1134. doi: 10.3390/ph19081134 (PMC13516065; doi:10.3390/ph19081134)
Supplement: Supplementary file 1 [file pharmaceuticals-19-01134-s001.zip › pharmaceuticals-4411030-supplementary.pdf]

## Supplementary File S1

## PRISMA 2020 Checklist Table

| Section / Item | Item No. | PRISMA 2020 Checklist Item Description | Reported and Content in the Manuscript | Location in manuscript |
|---|---|---|---|---|
| TITLE | | | | |
| **Title** | 1 | Identify the report as a systematic review. | Reported. The title explicitly identifies the nature of the study: "African Medicinal Plants Targeting Triple-Negative Breast Cancer (TNBC): A Systematic Review..." | Page 1 |
| ABSTRACT | | | | |
| **Abstract** | 2 | See the PRISMA 2020 for Abstracts checklist. | Reported. Includes background (TNBC aggressiveness), structured methods (PubMed, ScienceDirect, Scopus, Web of Science, AJOL), key results (30 genera, 22 families), and conclusions. | Page 1 |
| INTRODUCTION | | | | |
| **Rationale** | 3 | Describe the rationale for the review in the context of existing knowledge. | Reported. Section 1. Analyzes TNBC heterogeneity in Africa, healthcare economic barriers, limitations of current chemotherapies, and the reliance on traditional pharmacopeia. | Pages 2–3 |
| **Objectives** | 4 | Provide an explicit statement of the objective(s) or question(s) the review addresses. | Reported. Explicitly stated in the Abstract objective and the final paragraph of the Introduction: correlates traditional African ethnobotanical surveys with validated in vitro anti-TNBC cytotoxic efficacy, botanical taxonomy, and phytochemistry. | Pages 1, 3 |
| METHODS | | | | |
| **Eligibility criteria** | 5 | Specify the inclusion and exclusion criteria for the review and how studies were grouped for the syntheses. | Reported. Section 3.3. Specifies original peer-reviewed laboratory research, TNBC-specific cell models (MDA-MB-231, BT-20, HCC1937, 4T1), mandatory standard chemotherapy positive controls, publication window (2011–2026), and English language restriction. | Page 25 |
| **Information sources** | 6 | Specify all databases, registers, websites, organisations, reference lists and other sources searched. Specify the date when last searched. | Reported. Section 3.1. PubMed, ScienceDirect, Scopus, Web of Science, and AJOL were comprehensively searched. The final comprehensive query update was executed in February 2026. | Pages 23–24 |
| **Search strategy** | 7 | Present the full search strategies for all databases, registers and websites, including any filters and limits used. | Reported. Section 3.1. Full electronic boolean search equations combining botanical, oncological, and geographical terms are detailed for all repositories. | Pages 23–24 |
| **Selection process** | 8 | Specify the methods used to decide whether a study met the inclusion criteria (independent screening, automation tools). | Reported. Section 3.2. Initial screening and duplicate removal managed via Rayyan platform. Title/abstract screening and full-text assessment carried out independently by four reviewers (J.F.T.M., B.P.L., G.M.H., A.O.A.). | Page 24 |
| **Data collection process** | 9 | Specify the methods used to collect data from reports (independent extraction, automation tools). | Reported. Section 3.4. Data extraction was conducted using a standardized extraction matrix developed in Microsoft Excel, where two reviewers independently extracted the data, and any discrepancies were resolved by a third reviewer. | Pages 25–26 |
| **Data items** | 10a | List and define all outcomes for which data were sought. | Reported. Section 3.4. Outlines primary outcomes including anti-TNBC cell viability metrics (in vitro cytotoxicity, exact $IC_{50}$ threshold values, apoptosis induction markers, and transcriptomic indicators). | Pages 25–26 |
| | 10b | List and define all other variables for | Reported. Section 3.4. Outlines baseline secondary variables: full botanical name, family, local name, | Pages 25–26 |

| | | | | |
|---|---|---|---|---|
| | | which data were sought (botanical characteristics, funding, etc.). | geographical location, plant part used, mode of traditional preparation, extraction solvents, and isolated molecules. | |
| **Study risk of bias assessment** | 11 | Specify the methods used to assess risk of bias / methodological quality in the included studies. | Reported. Section 3.5. Internal quality criteria and methodological risk of bias were assessed independently by two reviewers across six domains, scoring each domain from 0 to 2 to reach a maximum quality score of 12. | Page 26 |
| **Effect measures** | 12 | Specify for each outcome the effect measure(s) used in the synthesis or presentation of results. | Reported. Section 3. Methodology & Results. Categorical distribution profiles (percentages for active families/genera, plant parts, and solvents) and raw quantitative experimental boundaries ($IC_{50}$) values expressed in μg/mL or μM) are utilized. | Pages 1, 4 |
| **Synthesis methods** | 13a | Describe the processes used to decide which studies were eligible for each synthesis. | Reported. Section 3. Methodology. Studies were systematically grouped and tabulated based on the presence of raw extract cytopharmacological metrics (Table 1) versus isolated pure secondary metabolites (Table 2). | Pages 23, 25-26 |
| | 13b | Describe any methods required to prepare the data for presentation or synthesis (data conversions). | Reported. Section 3.4 & Tables 1–2. Outlines chemical data logging protocols to tabulate heterogeneous experimental metrics, listing concentrations in standardized units (μg/mL for extracts, μM/nM for compounds). | Pages 6–12, 26 |
| | 13c | Describe any methods used to tabulate or visually display results of individual studies and syntheses. | Reported. Section 3. Methodology & Results. Describes visual tools used to display findings via comparative cytopharmacological matrices (Table 1, Table 2), a chemical structure canvas (Figure 1), and a pathway mechanism blueprint (Figure 2). | Pages 6–12, 14, 22 |
| | 13d | Describe any methods used to synthesize results and provide a rationale for the choice(s). | Reported. Section 3. Methodology. A narrative synthesis combined with qualitative pooling and tabular clustering was selected due to the high experimental and procedural heterogeneity of plant extract formulations. | Page 23 |
| | 13e | Describe any methods used to explore possible causes of heterogeneity among study results. | Reported. Section 3.5 & Results. Heterogeneity among reported activities was explored qualitatively by stratifying study features across specific assessment domains (botanical authentication, extraction solvent classes, cell lines, and replication metrics). | Pages 26–27 |
| | 13f | Describe any sensitivity analyses conducted to assess robustness of the synthesized results. | Reported. Section 3.5. Robustness of the overall evidence was addressed via a tailored 12-point quality score matrix to filter out or isolate low-quality studies (score < 6) from high-compliance literature (score ≥ 9). | Page 26 |
| **Reporting bias assessment** | 14 | Describe any methods used to assess risk of bias due to missing results in a synthesis (arising from reporting biases). | Reported. Section 3.5. Biases due to missing results were addressed by evaluating whether primary studies evaluated parallel toxicity on normal cell lines to report a Selectivity Index (SI), tracking the selective non-reporting of high-toxicity data. | Page 26 |
| **Certainty assessment** | 15 | Describe any methods used to assess certainty (or confidence) in the body of evidence for an outcome. | Reported. Section 3.5. Overall confidence in the anti-TNBC evidence was established through a quality categorization system, grading the body of literature into Low Risk (high quality), Moderate Risk, or High Risk (low quality) tiers. | Page 26 |
| **RESULTS** | | | | |
| **Study selection** | 16a | Describe the results of the search and selection process, ideally using a flow diagram. | Reported. Section 3.2. Outlines total flow from 1,300 database items down to 52 primary research papers (fully detailed in the embedded PRISMA Flow Diagram). | Pages 24-25 |
| | 16b | Cite studies that might appear to meet the inclusion criteria, but | Reported. Section 3.2. Detailed breakdown of the 108 excluded full-text articles, explicitly classified under pre-defined grounds (secondary literature, | Page 24 |

| | | | | |
|---|---|---|---|---|
| | | which were excluded, and explain why. | general non-TNBC lines like MCF-7, lack of controls, non-African flora, synthetic chemicals). | |
| **Study characteristics** | 17 | Cite each included study and present its characteristics. | Reported. Section 2. Results (Table 1 & Table 2). Methodological and biological baselines of all 52 studies are systematically cross-referenced and catalogued. | Pages 6–12 |
| **Risk of bias in studies** | 18 | Present assessments of risk of bias for each included study. | Reported. Section 2.4 & Table 4. Individual quality scores and specific risk allocations (+, ?, -) are detailed study-by-study for all included plant taxa. | Pages 12, 27 |
| **Individual results** | 19 | For all outcomes, present, for each study summary statistics and effect estimates. | Reported. Section 2 (Tables 1 & 2). Exact quantitative indicators ($IC_{50}$} raw bounds), assay types, exposure times, and targeted cancer lines are provided for each record. | Pages 6–12 |
| **Results of syntheses** | 20a | For each synthesis, briefly summarise the characteristics and risk of bias among contributing studies. | Reported. Section 2.4. Summarizes overall reporting compliance across the 52 studies, highlighting high compliance for extraction protocols but widespread gaps in botanical voucher deposition and normal cell line testing. | Page 12 |
| | 20b | Present results of all statistical syntheses conducted. | Reported. Section 2. Presents descriptive quantitative pooling, frequency distributions of the most active botanical families (Asteraceae, Fabaceae), and cluster tracking of $IC_{50}$ activity thresholds. | Pages 1, 4 |
| | 20c | Present results of all investigations of possible causes of heterogeneity among study results. | Reported. Section 2.1 & Section 2.2. Details comparative subgroup findings, demonstrating that non-polar organic solvent extracts (e.g., dichloromethane, chloroform) consistently yielded lower $IC_{50}$ values than crude aqueous preparations. | Pages 4, 7, 28 |
| | 20d | Present results of all sensitivity analyses conducted to assess the robustness of the synthesized results. | Reported. Section 2.4. Shows that the dominant anti-TNBC profile and therapeutic conclusions regarding key plant groups remained stable when isolating and comparing high-score papers against low-score literature. | Page 12 |
| **Reporting biases** | 21 | Present assessments of risk of bias due to missing results (arising from reporting biases) for each synthesis assessed. | Reported. Section 2.4. Evaluates the systematic non-reporting of negative data (inactive African plants) and details how the widespread lack of Selectivity Index (SI) reporting represents a critical source of selective outcome reporting bias. | Pages 6, 12 |
| **Certainty of evidence** | 22 | Present assessments of certainty (or confidence) in the body of evidence for each outcome assessed. | Reported. Section 2.4 & Section 2.5. Synthesizes overall confidence, showing high reproducible certainty for basic in vitro screening on MDA-MB-231 lines, but low experimental certainty regarding downstream molecular apoptotic targets. | Page 12 |
| **DISCUSSION** | | | | |
| **Discussion** | 23a | Provide a general interpretation of the results in the context of other evidence. | Reported. Section 2.5. Features a deep, structured mechanistic discussion organized by major secondary metabolite families (alkaloids, terpenoids, polyphenols) and prominent active plant taxa (Tulbaghia violacea, Annona muricata, Withania somnifera). | Pages 14–21 |
| | 23b | Discuss any limitations of the evidence included in the review. | Reported. Section 2.5. Criticizes the baseline weaknesses of the included primary literature: an over-reliance on basic in vitro screens, a complete absence of in vivo validation models, and a critical lack of standardized extract chemical fingerprints. | Pages 12, 28 |
| | 23c | Discuss any limitations of the review processes used. | Reported. Section 2.5 & Section 4. Discloses methodological limitations of the search protocol, including the strict English-language restriction, omission of French/Portuguese regional African journals, and exclusion of gray literature databases. | Page 24 |
| | 23d | Discuss implications of the results for practice, | Reported. Section 4 (Conclusion and Future Perspectives). Proposes clear operational roadmaps for future pharmacognostic validation, | Pages 2, 28 |

| | | | | |
|---|---|---|---|---|
| | | policy, and future research. | rigorous biochemical standardization, and the protection of African botanical biodiversity. | |
| **OTHER INFO.** | | | | |
| **Registration and protocol** | 24a | Provide registration information for the review, including register name and registration number. | Reported. Section 3.2. States that the search architecture and criteria were formally submitted to the International Prospective Register of Systematic Reviews (PROSPERO) and are awaiting standard ID allocation. | Page 24 |
| | 24b | Indicate where the review protocol can be accessed, or state that a protocol was not prepared. | Reported. Section 3.2. States that the systematic review protocol architecture was formally submitted to the International Prospective Register of Systematic Reviews (PROSPERO) for public archiving and administrative tracking. | Page 24 |
| | 24c | Describe and explain any amendments to information provided at registration or in the protocol. | Reported. Section 3.2. Explicitly confirms that no functional alterations, structural criteria changes, or workflow adjustments were made to the pre-registered review design during implementation. | Page 24 |
| **Support** | 25 | Describe sources of financial or non-financial support for the review, and the role of the funders. | Reported. Funding Section: Explicitly discloses institutional financial backing provided by the Universitat Ramon Llull Barcelona via dedicated project allocation (Grant number: 9713952 URL PDI 2026 NTUNGWE), which also fully covered the Article Processing Charges (APC) (p. 28). Role of Funders & Conflicts: In strict compliance with transparency protocols, the authors declare that the funders had no role in the study design, literature search, screening process, data extraction, qualitative/quantitative analysis, interpretation of ethnobotanical and cytotoxic insights, decision to publish, or preparation of the manuscript (p. 29). All authors maintain independent scientific control over the study and declare no conflicts of interest (p. 29). Non-Financial & Institutional Support: Beyond direct financial backing, this systematic review was actively supported by the infrastructural resources and academic facilities of the Department of Organic Chemistry at the University of Yaoundé I, the Department of Chemistry at the University of Douala, the Department of Chemistry at the University of Dschang, and the Department of Chemistry at the University of Maroua (p. 29). Additionally, analytical validation and collaborative tools were facilitated via external research support extended by TÜBITAK-BİDEB, TWAS, and UNESCO (Grant No. 3240338187) (p. 29). | Page 28-29 |
| **Competing interests** | 26 | Declare any competing interests of review authors. | Reported. Conflicts of Interest Section. Contains a formal compliance statement confirming that all participating authors have zero personal, financial, or institutional conflicts to declare. | Page 29 |
| **Availability of data...** | 27 | Report which materials are publicly available and where they can be found (forms, extracted data, code). | Reported. Data Availability Statement. Confirms that all template data collection forms, data extraction matrices, and baseline taxonomic logs are fully available to the public within the manuscript's Supplementary Files. | Page 28 |
